# Gene Expression and Co-expression Networks Are Strongly Altered Through Stages in Clear Cell Renal Carcinoma

**DOI:** 10.3389/fgene.2020.578679

**Published:** 2020-11-03

**Authors:** Jose María Zamora-Fuentes, Enrique Hernández-Lemus, Jesús Espinal-Enríquez

**Affiliations:** ^1^Computational Genomics Division, National Institute of Genomic Medicine, Mexico City, Mexico; ^2^Centro de Ciencias de la Complejidad, Universidad Nacional Autónoma de Mexico, Mexico City, Mexico

**Keywords:** clear cell renal carcinoma, gene co-expression networks, SLC6A19 progressive underexpression, PLG progressive underexpression, cancer progression stages, SAA2-SAA4 progressive overexpression, CXCL13 progressive overexpression, loss of long-range co-expression

## Abstract

Clear cell renal carcinoma (ccRC) is a highly heterogeneous and progressively malignant disease. Analyzing ccRC progression in terms of modifications at the molecular and genetic level may help us to develop a broader understanding of its patho-physiology and may give us a glimpse toward improved therapeutics. In this work, by using TCGA data, we studied the molecular progression of the four main ccRC stages (i, ii, iii, iv) in two different yet complementary approaches: (a) gene expression and (b) gene co-expression. For (a) we analyzed the differential gene expression between each stage and the control non-cancer group. We compared the progression molecular signature between stages, and observed those genes that change their expression patterns through progression stages. For (b) we constructed and analyzed co-expression networks for the four ccRC progression stages, as well as for the control phenotype, to observe whether and how the co-expression landscape changes with progression. We separated genomic interactions into intra-chromosome (*cis-*) and inter-chromosome (*trans-*). Finally, we intersected those networks and performed functional enrichment analysis. All calculations were made over different network sizes, from the top 100 edges to top 1,000,000. We show that differential expression is quite similar between ccRC progression stages. However, interestingly, two genes, namely SLC6A19 and PLG show a significant progressive decrease in their expression according to ccRC stage, meanwhile two other genes, SAA2-SAA4 and CXCL13 show progressive increase. Despite the high similarity between gene expression profiles, all networks are substantially different between them in terms of their topological features. Control network has a larger proportion of *trans-* interactions, meanwhile for any stage, the amount of *cis-* interactions is higher, independent of the network cut-off. The majority of interactions in any network are phenotype-specific. Only 189 interactions are shared between the five networks, and 533 edges are ccRC-specific, independent of the stage. The small resulting connected components in both cases are formed by genes with the same differential expression trend, and are associated with important biological processes, such as cell cycle or immune system, suggesting that activity of these categories follows the differential expression trend. With this approach we have shown that, even if the expression program is similar during ccRC progression, the co-expression programs strongly differ. More research is needed to understand the delicate interplay between expression and co-expression, but this is a first approach to enclose both approaches in an integrative view aimed at a deeper understanding in gene regulation in tumor evolution.

## 1. Introduction

The term *renal cell cancer* refers to a heterogeneous group of cancers derived from renal tubular cells. In the last years, pathology-based and basic cancer research programmes have characterized different renal tumor entities (Moch, 2013). Renal cell carcinoma is a group of malignancies arising from the epithelium of the renal tubules (Moch, 2013). Renal cancer may be seen as several histologically defined cancers. Those present different genetic drivers, epigenetic marks, clinical courses, and also therapeutic responses (Ricketts et al., 2018).

Histologically, renal cancer has been divided into three major subtypes, clear cells, papillary renal cell carcinoma, and chromophobe renal cell carcinoma (Moch et al., 2016). Clear cell renal cell carcinoma (ccRC) is the most common subtype (≈75%); papillary renal cell carcinoma (PRCC) accounts for 15–20% and is subdivided into types 1 and 2; and chromophobe renal cell carcinoma (ChRCC) represents ≈5% of renal cell carcinomas (Jaffe et al., 2001; Moch et al., 2016).

Molecular and genomics characterization of these tumors have been conducted elsewhere. For instance, the Cancer Genome Atlas Consortium (TCGA, The Cancer Genome Atlas Research Network, 2013, 2016) has provided the most common deregulated processes in kidney cancer in general (The Cancer Genome Atlas Research Network, 2013), as well as in ccRC in particular (The Cancer Genome Atlas Research Network, 2016). Events such as Krebbs cycle downregulation, upregulation of pentose phosphate pathway genes or important genomics rearrangements in TERT region have been observed as recurrent deregulated processes.

Inside the ccRC subtype, particular subgroups have been identified. Such subgroups have been related to epigenetic modifications, somatic mutations, or genomic rearrangements within the TERT promoter region (Ricketts et al., 2018). Proteins associated with Warburg effect, as well as molecular predictors of late stage (Neely et al., 2016), have also been associated to ccRC. Several references regarding mutations of von Hippel-Landau (VHL) tumor suppressor gene have also been reported (Kaelin, 2004; Cowey and Rathmell, 2009; Arjumand and Sultana, 2012).

Regarding epigenetic modifications, comprehensive revisions have reported an increasing number of them (see Jung et al., 2009; Redova et al., 2011; Li et al., 2015). For instance, for ccRC, miR-99a, miR-106a, miR-125b, miR-144, miR-203, miR-378, or mir-28-5p have shown a dual behavior, oncogenic and oncosuppressive (Wang et al., 2016; Braga et al., 2019). Genes such as the aforementioned VHL, or RASSF1A, CDH1, and APAF1 have been found to be susceptible to hypermethylation (Dmitriev et al., 2014; Braga et al., 2015).

Despite all those advances in characterizing molecular features of renal cancer, histo-pathological aspects still contain crucial information for accurate clinical interventions. In those terms, progression stages (according to the Gold standard reference in cancer staging, Edge et al., 2010) provide us important elements to have, in combination with molecular characteristics, a broader and more integrative point of view regarding renal cancer. Hence, understanding progression in terms of molecular and genetic factors could help us to understand the disease with higher accuracy.

In this work, we used information from molecular and histo-pathological factors to unveil specific characteristics that change during progression stages. To this end, we focused on the molecular progression of clear cell Renal carcinoma (ccRC) by two different yet complementary approaches: (a) gene expression and (b) gene co-expression. For (a) we analyzed the differential expression of all genes at the four progression stages vs. the control non-cancer group, and between stages, to observe the gene expression pattern for each progression stage. We compared the progression signature between stages, and observed whether or not a set of genes change their expression patterns through progression stages.

For (b), we constructed and analyzed co-expression networks for the four ccRC progression stages, as well as for the control phenotype and compared between them, in order to have a quantitative indicator to distinguish and observe whether or not the co-expression landscape changes progressively.

In previous works from our group, we observed abrupt changes in the way that genes co-express: for instance, we have documented a substantial decrease of inter-chromosome (*trans-*) gene-gene interactions in breast cancer (Espinal-Enriquez et al., 2017; Dorantes-Gilardi et al., 2020; García-Cortés et al., 2020). We decided to separate gene-gene interactions into intra-chromosome (*cis-*) and inter-chromosome (*trans-*). We performed functional enrichment analyses for each whole-network, and also by communities inside networks, by assuming that network structure may guard functional features of an oncogenic phenotype (Alcalá-Corona et al., 2016, 2017, 2018; Hernández-Lemus et al., 2019).

We wanted to quantify similarities and differences between consecutive progression stages, since with this information one may isolate those features that are conserved or change between one stage to the following. To do so, we obtained the network intersections and differences between consecutive progression phenotypes, starting with Stage I vs. Control network, Stage II vs. Stage I, etc. In a complementary task, we intersected the five networks (the four stages and control) to observe which genes and interactions are conserved throughout all phenotypes. Additionally, we intersected the four progression stage networks to observe those interactions that appear in cancer but are not present in a healthy phenotype. The resulting networks were then analyzed via over-representation analysis. We observed those processes involved in the resulting networks and also the respective differential expression patterns.

## 2. Materials and Methods

**Figure 1:**
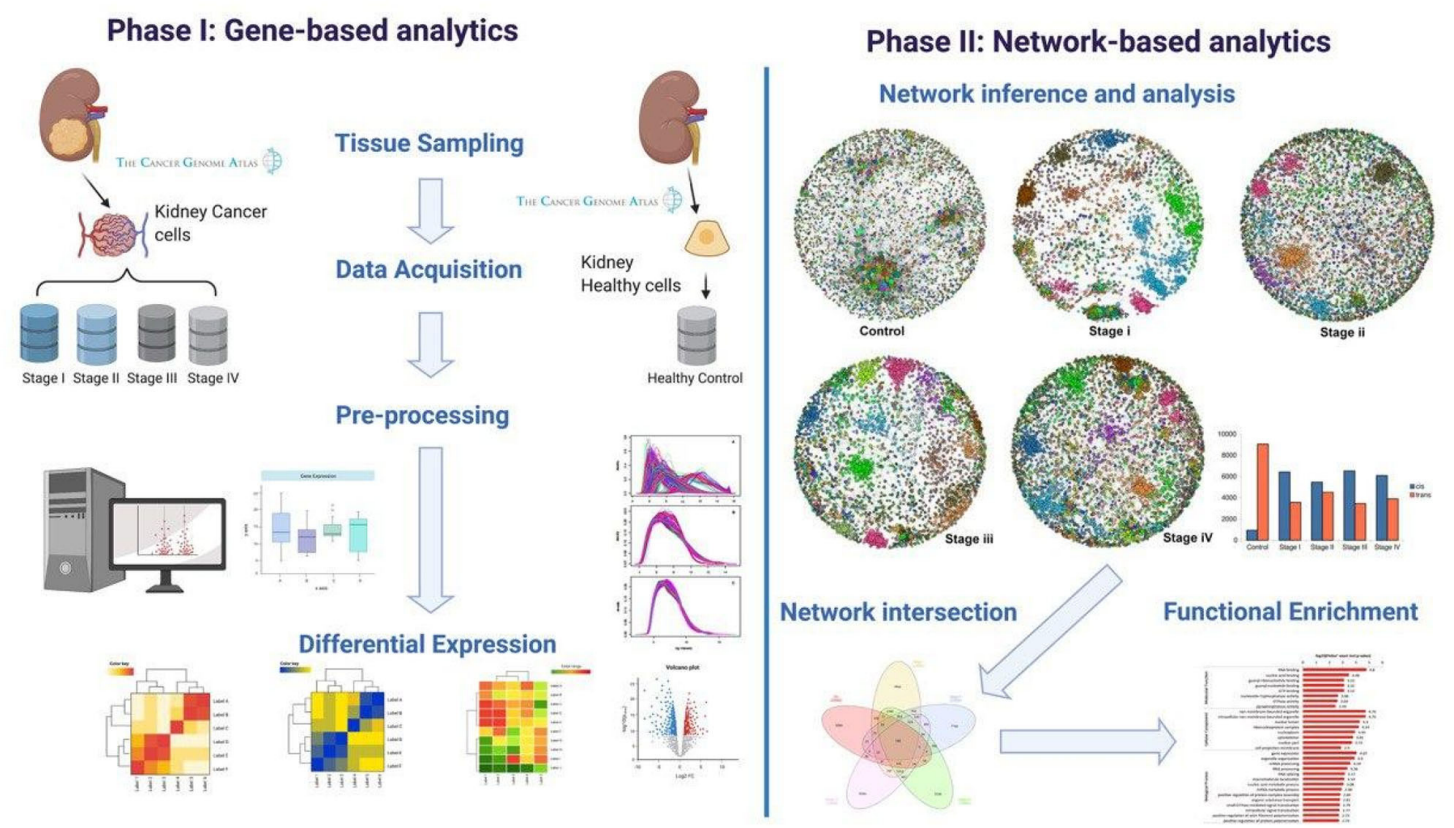
Workflow. Graphical representation of the computational pipeline performed here. (**Left**) Gene-based analysis. (**Right**) Network-based analyses.

A graphical representation of our methodology can be found in Figure 1. Our workflow can be broadly divided into two main branches: gene-based and network-based analyses. These in turn, can be divided into four main steps: (1) Data acquisition, (2) Pre-processing, (3) High-level processing, and (4) Functional enrichment.

### 2.1. Data Acquisition

We obtained the complete dataset from GDC clear cell renal carcinoma repository (https://portal.gdc.cancer.gov/repository). For this purpose, we developed a set of scripts that uses as input the TCGA project transcriptomic data and metadata (in this case, ccRC). The scripts collect all transcriptome profiling samples, as well as clinical data available for the same samples. The RNA-seq transcriptomic profiles were pruned, keeping those genes with valid numeric values and its associated ENSEMBL ID.

Tumor samples were separated into stages according to the *tumor_stage* variable, provided by TCGA for each clinical file. In the case that *tumor_stage* value was *not reported*, we decided to discard that sample.

We used RNA-Seq level 3 gene expression files from The Cancer Genome Atlas from 608 ccRC samples. We divided these patients by cancer progression stage, as well as control non-tumor tissue. Number of cases for each stage is shown in Table 1.

**Table 1:** RNA-Seq data from ccRC patients per progression stage.

**Tissue**	**Control**	**Stage I**	**Stage II**	**Stage III**	**Stage IV**
ccRC	72	272	59	123	82

### 2.2. Data Pre Processing

We carried out a data pre-processing pipeline in three phases. (1) pre-normalization quality control, (2) batch and bias corrections (normalization) and (3) post-normalization quality control. Data pre-processing was conducted as previously (Drago-García et al., 2017; Espinal-Enriquez et al., 2017; de Anda-Jáuregui et al., 2019b,c; García-Cortés et al., 2020; Serrano-Carbajal et al., 2020). Briefly, we assessed (a) biotype abundances, to assure that samples contained protein coding genes. (b) gene counts expression boxplots were also evaluated per biotype to confirm that the highest median expression corresponded to protein coding genes. (c) Finally, we evaluated the number of detected genes per sample, by using saturation plots. These steps were performed with standard R package *NOISeq* (Tarazona et al., 2011). Normalization method for correct Length bias (full) and GC content (full) was Within-lane. Additionally we applied a “TMM” normalization to eliminate RNA composition biases between libraries and prepare data to find Differentially Expressed Genes. Risso et al. (2011). PCAs and plots are shown in Supplementary Material 1. Genes were filtered by mean expression values (*mean* > 10). Normalization to correct batch effect was performed by using ARSyN (Nueda et al., 2012) implemented in NOISeq package. Scripts to perform pre-processing analysis can also be found at https://github.com/josemaz/kidney-stages.

### 2.3. Differential Expression

Differential gene expression analysis was performed to compare gene expression between each ccRC stage vs. control. This analysis was performed via empirical Bayes moderation of the standard errors using *edgeR* package (Robinson et al., 2010). To consider a gene as differentially expressed, we considered a Log_2_Fold Change (|*LFC*| > 2.0) cut-off.

#### 2.3.1. Statistical Significance and Multiple Hypothesis Testing

To account for multiple comparisons of gene profiles, we implemented Benjamini & Hochberg False Discovery Rate correction calculations. The FDR-adjusted *p*-value cut-off was set to be 0.05 for each comparison.

We also performed a multi-group comparison based on Likelihood ratio test (LRT) method to obtain all group contrasts (Love et al., 2014). With this method, implemented in the DEseq2 R package, we used the deviation of each group in the calculation of *p*-values for every contrast. We filtered genes with a corrected *p*-value less than 0.05 and log-fold change −0.5 > |*LFC*| > 0.5 for each contrast. This last, searching for differentially expressed genes, not only between genes of cancer stages and control samples, but also between stages.

Since ccRC data is separated into stages, we observed those genes that change in agreement with the stages, i.e., differential expression increases or decreases progressively with stages. To determine the significance of those differences, we performed a Wilcoxon signed rank test between individual gene expression at different stages.

### 2.4. Network Analysis

We used the mutual information (MI) statistical dependence measure to quantify co-expression between genes. We used the MI implementation on the ARACNe algorithm (Margolin et al., 2006), as previously described (Alcalá-Corona et al., 2017, 2018; Espinal-Enriquez et al., 2017; de Anda-Jáuregui et al., 2019c; García-Cortés et al., 2020), to determine all gene-gene interactions in the genome for the four ccRC stages and for control networks. With this procedure we inferred five networks, one for each stage and one for the control phenotype.

#### 2.4.1. Network Interactions Assessment

In order to have those interactions with a higher relevance (as given by their mutual information values) for each phenotype, and in view of the so-called network sparsification problem, (determination of the number of *significant* edges that represent better the network structure consistent with the data), we decided to perform network cut-offs spanning over several scales well above and well beyond our working thresholds to account for possible size-effects. The cut-off thresholds range from the top 100 interactions, to the top 1,000,000 interactions, i.e., five orders of magnitude in network size. We performed those cut-offs to assess whether the effects under study, such as in the *cis-* rates was indeed due to network size.

Network visualizations were performed using *Cytoscape V 3.8.1* (Shannon et al., 2003), as well as the *iGraph* Python library (Csardi and Nepusz, 2006).

Since a relevant question underlies on whether in these networks, the effect of loss of *trans-* co-expression was also lost as in breast cancer (Espinal-Enriquez et al., 2017; de Anda-Jáuregui et al., 2019a,b,c; Dorantes-Gilardi et al., 2020; García-Cortés et al., 2020), we separated co-expression interactions into *cis-* (intra-chromosome), and *trans-* (inter-chromosome). We observed the *cis-/trans-* ratio for each phenotype.

### 2.5. Stages Intersections

One of the most important issues that might be addressed with a dataset such as the one we have, by means of the methodology exposed here, is how the co-expression landscape is modified throughout cancer progression. Derived from the latter, we compared the differences and intersections between the control network, and each progression stage. First, we observed the differences between network interactions, i.e., those gene-gene interactions that are not shared between phenotypes. Concomitantly, we observe those genetic interactions shared between control network and any ccRC progression stage.

Additionally, a question derived from the latter, is which interactions are conserved between all phenotypes, and also important, between cancer stages only. For that purpose, we performed a multi-group intersection to obtain the sub-network integrated by those links shared by all phenotypes, and also the ccRC-only sub-network.

### 2.6. Functional Enrichment

Functional enrichment analysis was performed using the *g:profiler* (Raudvere et al., 2019) API for Python. *g:Profiler* uses the hypergeometric test to measure the significance of a functional term in the input gene list (Reimand et al., 2007, 2011, 2016). Multiple testing corrections were performed by the g:SCS algorithm as implemented in *g:Profiler* with significance level *a* = 0.05; and a False Discovery Rate of 0.05.

It is worth noticing that in order to consider the network structure in the functional enrichment, the g:SCS algorithm was implemented over network communities, and not over the whole networks. For community detection in networks we performed the *Infomap* algorithm (Rosvall and Bergstrom, 2008), as implemented in Alcalá-Corona et al. (2016), Alcalá-Corona et al. (2017), and Alcalá-Corona et al. (2018).

In order to provide a clear and easy-to-follow manner to reproduce the results reported here, the five expression matrices, and all code for developing this work are provided in https://github.com/josemaz/kidney-stages. In this repository it can be found the code to reproduce all results, since the data download until functional enrichment.

## 3. Results and Discussion

### 3.1. Differential Expression Is Similar Between ccRC Stages

After *low-level processing* of the four tumor stage data and control samples, we performed differential expression analysis for each stage compared with control samples (Supplementary Material 2).

**Figure 2:**
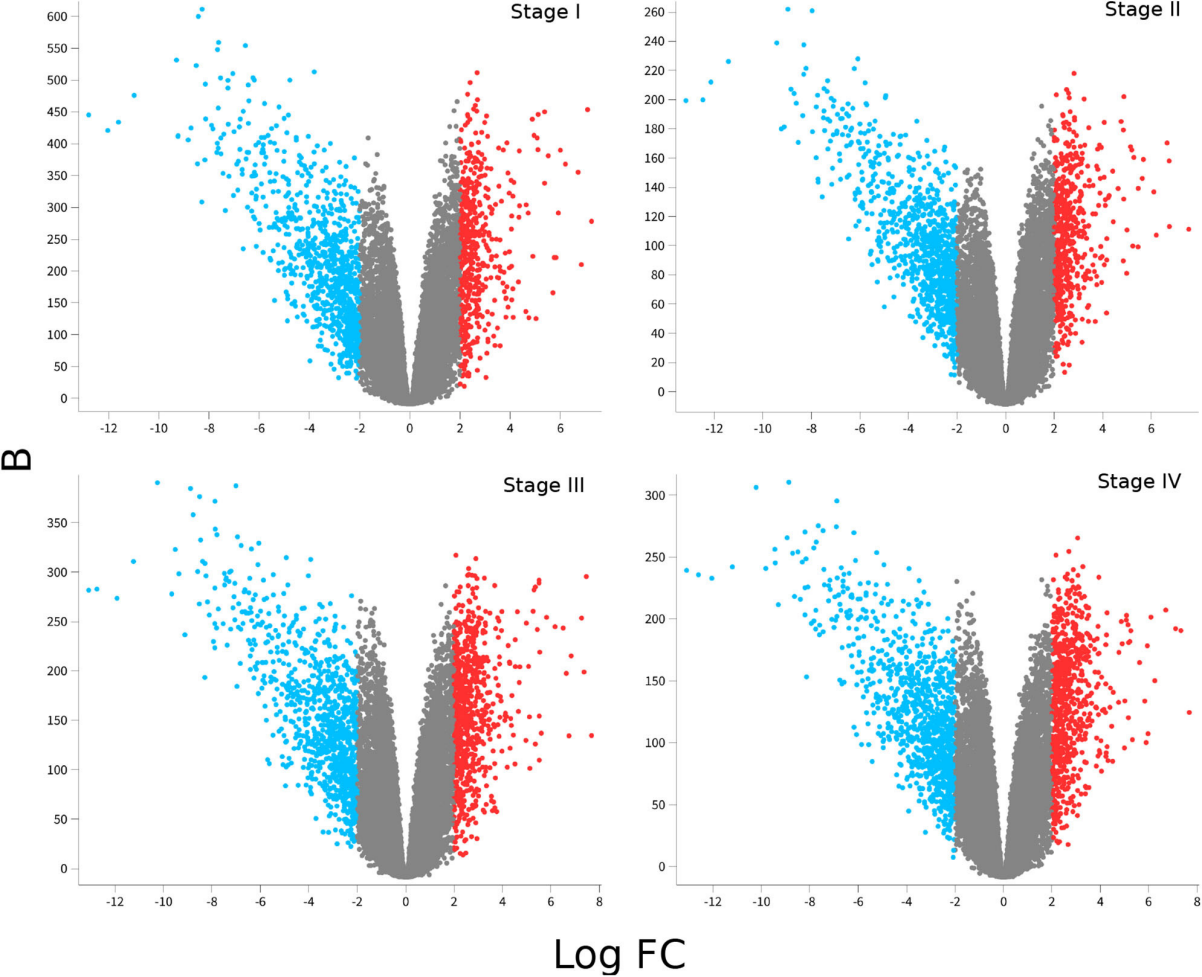
Differential gene expression for each ccRC stage. In these volcano-plots, the differential expression between each stage vs. control samples is depicted. Red dots represent overexpressed genes, meanwhile underexpressed ones are in blue. Notice that underexpressed genes are more broadly distributed than overexpressed ones, and *Log*_2_*FC* values are similar in the four figures; however, B-statistics change depending on the ccRC stage.

Figure 2 shows volcano plots for differentially expressed genes in the four stages. Large similarity in the distribution of genes and range of values for the four stages is visible. The rank of differentially expressed genes is also similar. Table 2 shows the Spearman's correlation of ranks between the four stages. As it can be observed, Spearman's ρ_*corr*_ > 0.948 in all cases, evidencing the similitude between differentially expressed gene ranks.

**Table 2:** Spearman correlation between rank of differentially expressed genes for all stages.

**ccRC stage**	**Stage I**	**Stage II**	**Stage III**	**Stage IV**
Stage I	1	0.995	0.974	0.948
Stage II	0.995	1	0.995	0.958
Stage III	0.974	0.994	1	0.997
Stage IV	0.948	0.958	0.997	1

#### 3.1.1. SLC6A19 and PLG Genes Show Progressively Decreasing Expression

**Figure 3:**
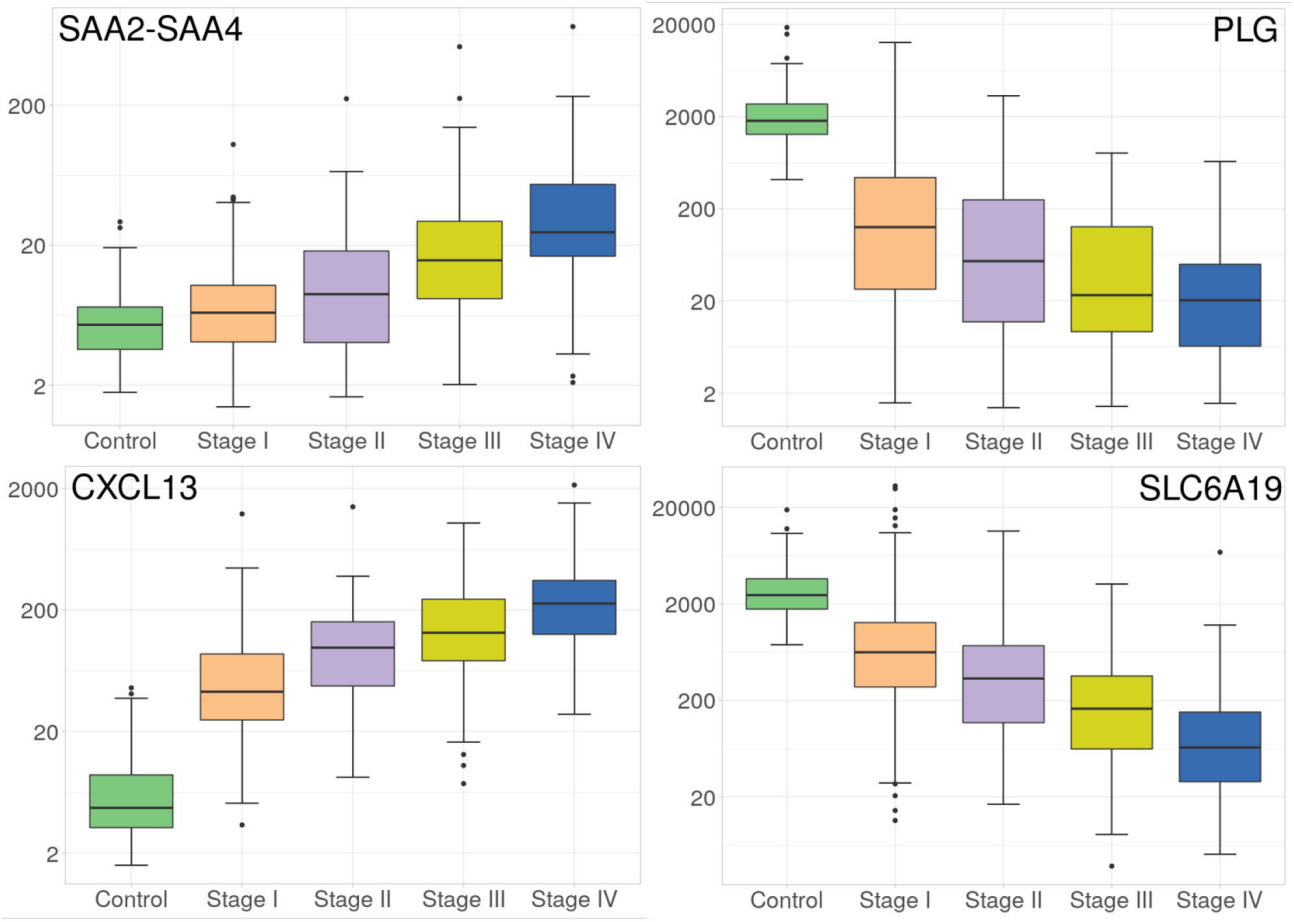
Progressive increase and decrease in expression of four genes at the different ccRC stages. These barplots show the average expression of SAAC2-SAAC4 and CXCL13 genes **(left)**, and SLC6A19 and PLG genes **(right)**. Different colors represent the progression stages. Notice that the Y axis (gene expression) is, in all cases, depicted in log scale.

Despite the fact that the four volcano plots are similar, and Spearman's correlation between all stages is high, some genes appear to be expressed according to tumor progression stages, such as the case of genes observed in Figure 3. Interestingly, SLC6A19 and PLG, both show a remarkable decrease in their expression during progression stages (Figure 3).

#### 3.1.2. SAAC2-SAAC4 and CXCL13 Genes Show Progressively Increasing Expression

Now regarding the overexpression of genes during ccRC progression, we found that only two genes, namely SAAC2-SAAC4 and CXCL13 genes, are overexpressed according to tumor progression stages, as it can be observed at the left side of Figure 3. It is worth to note that in the four cases, those genes are differentially expressed between control and any stage, but also between consecutive stages. This result may have clinical relevance since these protein-coding genes may be used as biomarkers of clear cell renal carcinoma progression.

Furthermore, we conducted a multi-group differential expression analysis, to observe whether or not said difference in gene expression also appeared between stages. In all cases, these genes are differentially expressed. However, between stage III and IV, the *Log*_2_*FC* was set to 0.5. This means that the expression values of the four genes is different but not as largely different as in the previous stages. This could be due to the clinical and histo-pathological features that both stages may share.

To the best of our knowledge, the SLC6A19 gene has not been previously reported as importantly underexpressed in renal cancer, however, in the Human Protein Atlas, SLC6A19 underexpression has been reported as a biomarker for renal cancer (https://www.proteinatlas.org/ENSG00000174358-SLC6A19/pathology). SLC6A19 is highly expressed in kidney tissue (Fagerberg et al., 2014). Hence, its underexpression may bring relevant functional consequences.

PLG gene also presents a remarkable decrease throughout stages advance (Figure 3). Previously, PLG has been reported has decreased and a possible biomarker for renal carcinoma (Luo et al., 2018; Zhang et al., 2020).

In the case of CXCL13 overexpression, recently (Jiao et al., 2020), it has been found to be related to tumor-infiltrating immune cells, as well as bad prognosis in ccRC. In our case, we not only found the gene overexpresssed, but also progressively increased through the four stages.

Regarding SAA2-SAA4 gene, its overexpression has been observed as *unfavorable* in renal cancer, but at the same time *favorable* in breast cancer (https://www.proteinatlas.org/ENSG00000255071-SAA2-SAA4/pathology). SAA2-SAA4 is a naturally-occurred fusion between two serum amyloid genes (A2 and A4). SAA2-SAA4 overexpression has also been associated in metastatic brain tumor derived from papillary thyroid carcinoma (Schulten et al., 2016). It also has been associated with liver metastasis from colorectal tumor (Sayagués et al., 2016). The fact that SAA2-SAA4 overexpression has been associated with metastasis from neighboring primary tumor is matter of further research. However, it is worth mentioning that expression of this gene is progressively increased through ccRC progression stages.

To our knowledge, this is the first time that expression of SAA2-SAA4, CXCL13, PLG, and SLC6A19 have been reported to be differentially expressed through progression stages in clear cell renal carcinoma, showing a possible novel line of research related with ccRC genomic progressive alterations.

### 3.2. Control Network Is Topologically Different to Any Tumor Network

We found that all networks are substantially different among them, but the control one presents a more striking difference in terms of its topological features. Control network has a larger proportion of *trans-* interactions, whereas for any cancer stage the amount of intra-chromosome (*cis-*) interactions are more abundant than *trans-* ones.

**Figure 4:**
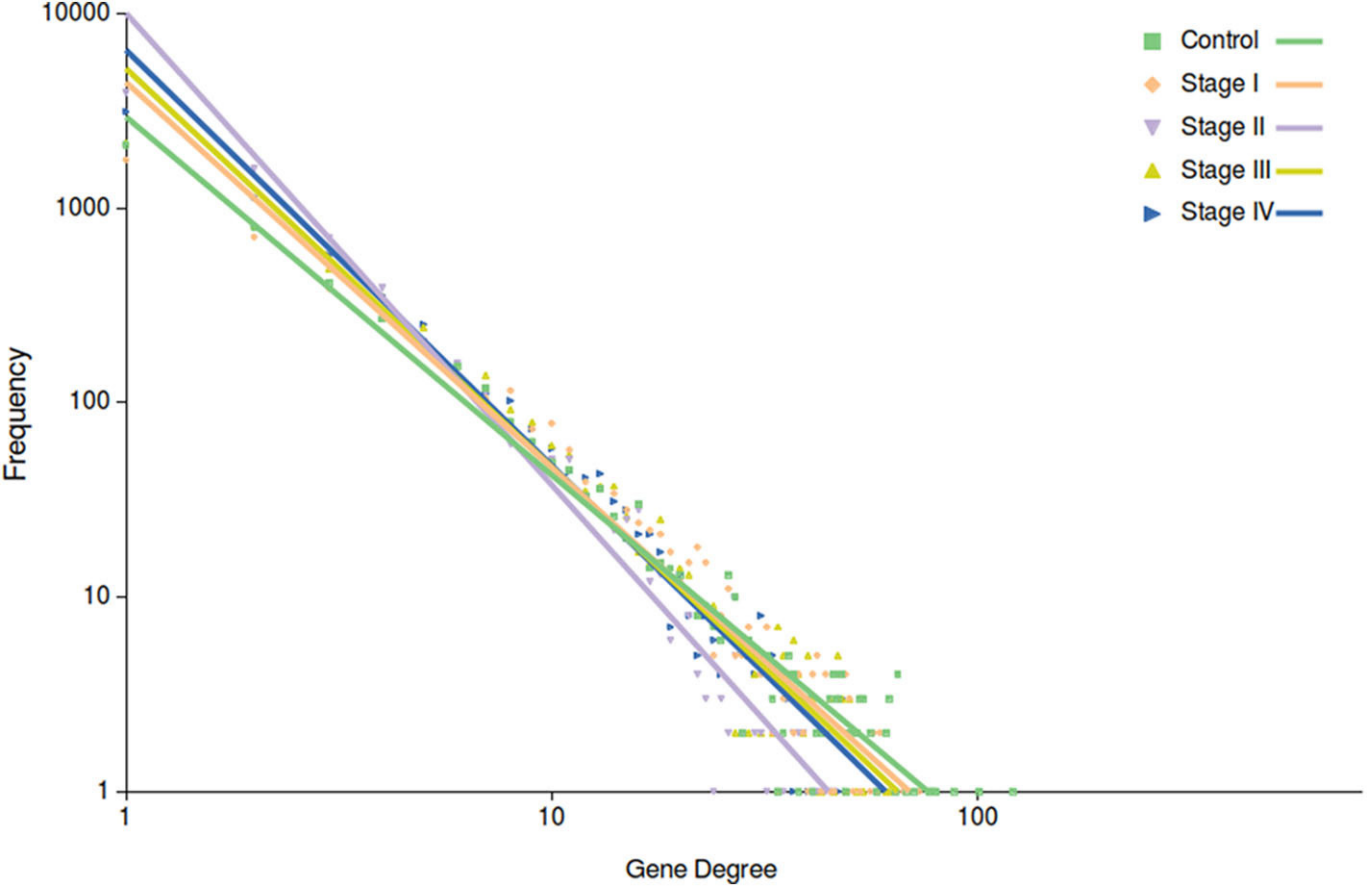
Degree distribution of the five networks. In this plot, points correspond to the degree distribution for each phenotype. Color code is the same than Figure 3. Curve fitting (*y* = *ax*^*b*^) to each degree distribution is also depicted. Notice that control network distribution slope (light green) is the lowest one.

Among the most important network parameters to examine is the degree distribution *p*(*k*). It is well-known that the *k* vs. *p*(*k*) plot and its parameters for curve fitting may reflect several properties related to the system itself. In the case of top 10,000 edges cut-off, we may observe that in all cases the distribution is well-fitted to a power law distribution (*y* = *ax*^*b*^). The differences are observed in Figure 4, in the different slopes of the curve fittings, as well as at the parameter level. The slope of control network degree distribution (light green) is the lowest one (−1.842), compared to the ccRC stages. Table 3 contains the parameters for the non-linear curve fitting of the five networks. The latter may describe that *long-range communication* is a feature in a healthy phenotype.

**Table 3:** Parameters of the non-linear curve fitting in all networks for the top 10,000 interactions.

**Parameter**	**Control**	**Stage I**	**Stage II**	**Stage III**	**Stage IV**
a	2941.8	4430.5	10047	5215.8	6490.8
b	−1.842	−1.982	−2.4266	−2.052	−2.137
Correlation	0.992	0.981	0.973	0.98	0.987
R-square	0.935	0.931	0.953	0.939	0.959

### 3.3. Statistical Networks Differences

#### 3.3.1. There Is a Preferential *cis-* Co-expression in ccRC Networks

**Figure 5:**
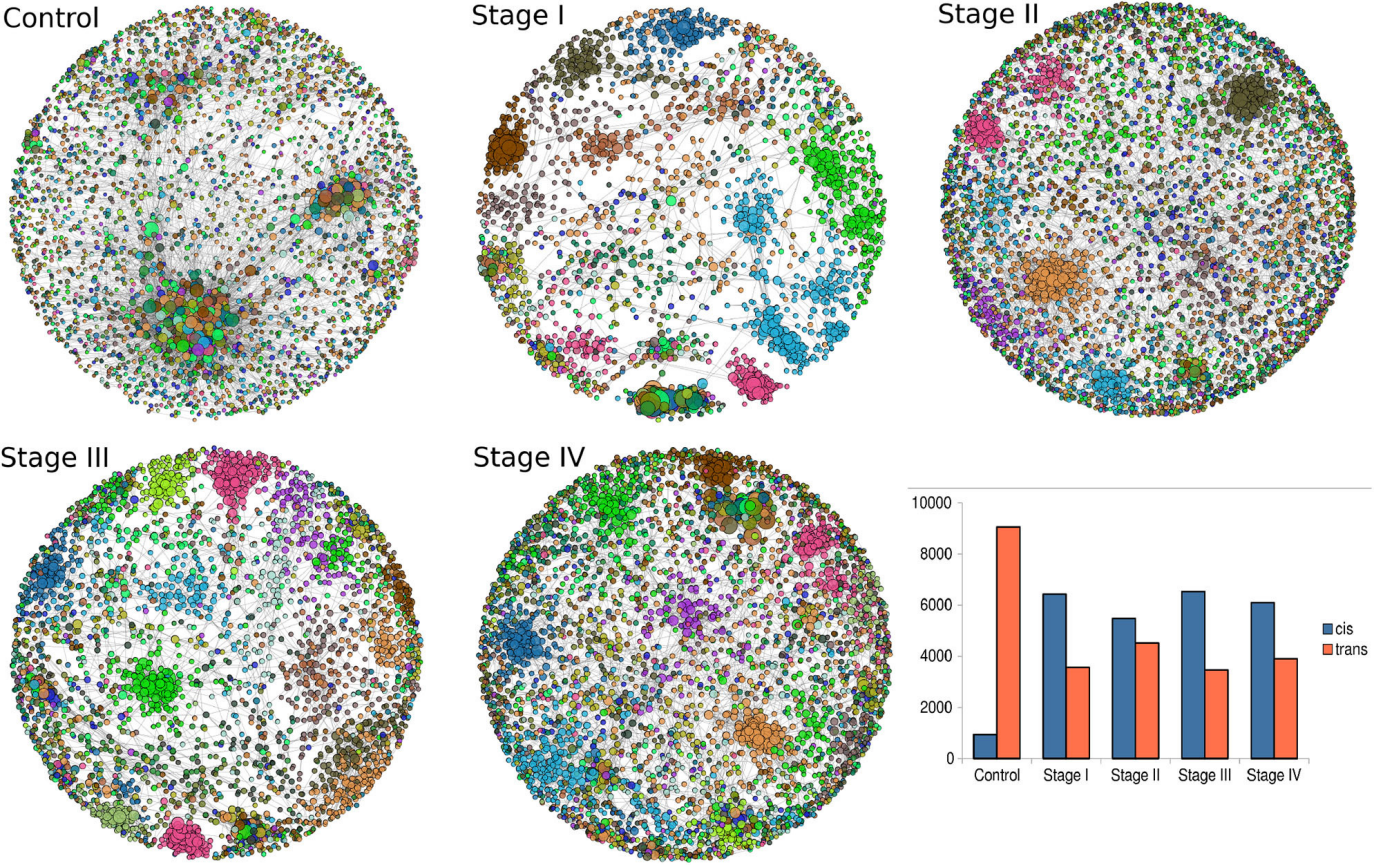
Network topologies of ccRC per stage. Top to bottom figures correspond to the largest connected component of control, stage I, stage II, stage III, and Stage IV, respectively. Color of nodes correspond to the chromosome to which each gene belongs to. The bar-chart represents the proportion of *cis-* (blue) and *trans-* (orange) interactions.

Giant connected components of each network are depicted in Figure 5. Genes are colored according the chromosome each gene belongs to. In the control network, genes co-express with genes from any chromosome, with a high prevalence of *trans-* interactions. Conversely, for the ccRC stages, in all cases there is preferential *cis-* co-expression. This is also reflected in the bar-charts at the bottom right part of Figure 5. Orange bars represent the number of *trans-* interactions, meanwhile *cis-* links are represented by blue bars.

#### 3.3.2. *cis-/trans-* Ratios Do Not Re-trace Progression Stages

In previous works from our group (García-Cortés et al., 2020), we have shown that *cis-/trans-* ratio increased with severity of breast cancer subtypes, being Luminal A, Luminal B, HER2+ and Basal the order of *cis-/trans-* ratios. There, we also shown that breast control network is the only graph that contains more *trans-* interactions than *cis-* ones.

Intuitively, one may expect (based on our previous experience with breast cancer) a progressive decrease in the number of *trans-* interactions, starting from the largest number in control network, decreasing throughout ccRC stages. However, this is not the case, as it can be also appreciated from the bar-charts, as well as from networks. The ccRC network with less *trans-* co-expression links is stage III, followed by stage I, stage IV, and finally stage II. However, the difference between control and any stage is also evident.

#### 3.3.3. Chromosome-Specific *cis-* Rates Are Different Between Phenotypes

**Figure 6:**
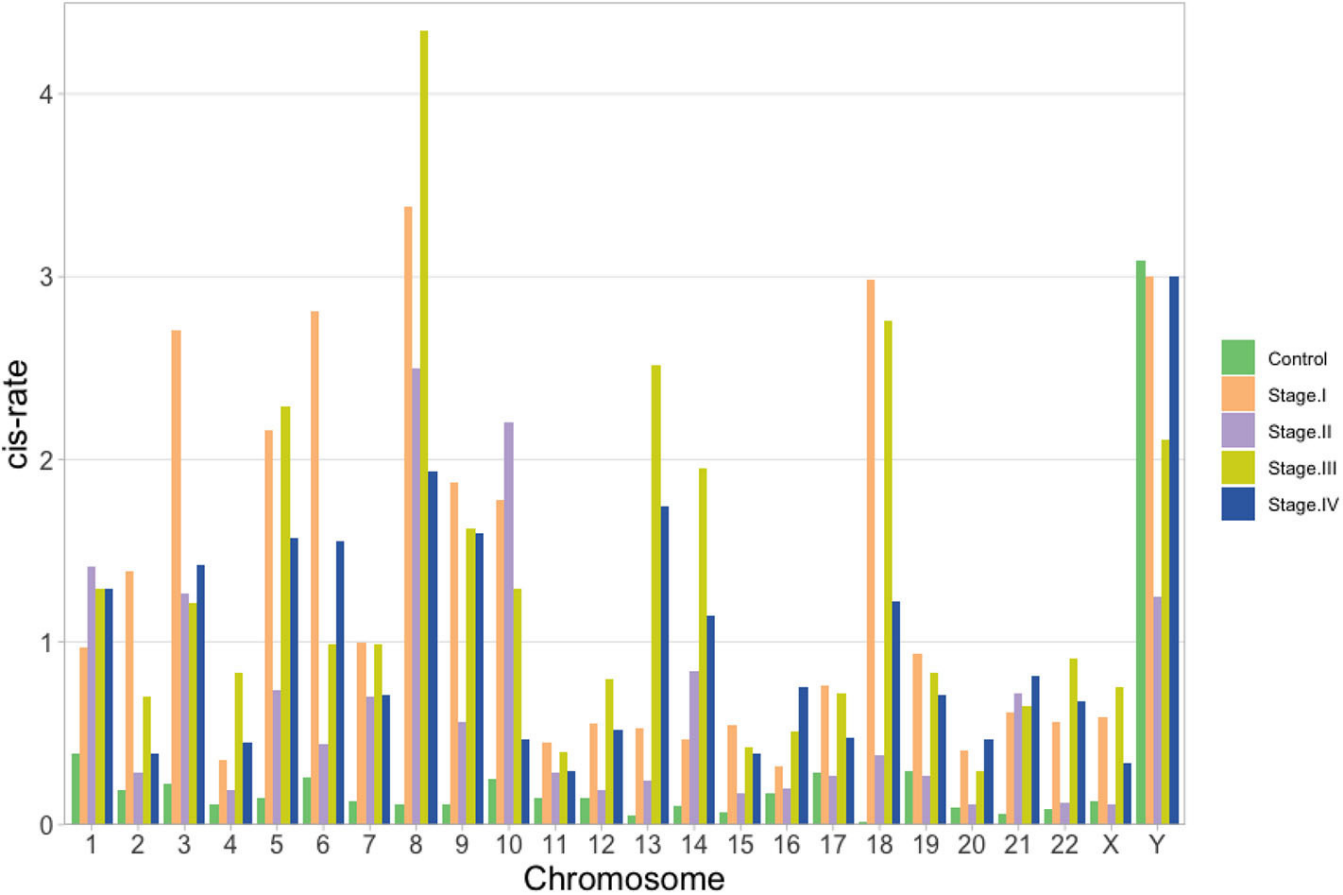
*cis-* rate (*cis edges/# of genes*) per chromosome for the five networks: green, orange, violet, yellow and blue for control, stage I, stage II, stage III, and stage IV, respectively. In all cases but for ChrY, the ratio is lower than 1 for the control network.

Once the proportion of global *cis-/trans-* interactions were obtained, isolated chromosome *cis-* rates were calculated. We defined the *cis- rate* as the number of *cis-* edges divided by the total number of edges in each network. As it can be observed in the barplot of Figure 6, for the control network, all chromosomes but ChrY have a *cis* − *rate* < 1, but in the case of Chr Y, all phenotypes have a *cis-* rate > 1. In general, stage III network has the highest *cis-* rates at the chromosome level.

### 3.4. Topological Differences Do Not Follow Progression Stages

As a first approach, network cut-off was set to top-10,000 edges, ranked by MI values. Each network contains a different number of genes. Since these networks are obtained from gene expression of kidney tissue, one may naively expect similarities in terms of genes and even interactions. Additionally, given that the networks under study were separated into progression stages, it also would be expected that consecutive stages were more similar between them than with the rest of networks.

In **Figure 8**, we show the number of shared interactions between phenotypes, as well as their differences. As expected, control network is the most different in terms of number of shared links with the ccRC networks. The percentage of divergence is 94% in the more similar case (stage I).

ccRC networks also differ vastly between them, more than 60% difference in any case. Stage II network is the most different, in terms of number of shared edges. Conversely, stage I and stage III networks are the more similar pair, even stage I and stage IV keep more shared edges between them (74%) than with stage II.

The latter results is surprising, taking into account the high similitude in terms of differential gene expression in the four phenotypes (Table 2). Network topologies and the concomitant co-expression programs do not coincide with the gene expression signatures of ccRC progression stages.

Additionally, the small number of shared genomic interactions between control and ccRC networks also reflects, a radical rearrangement of the transcriptional program between health and disease.

Biologically, the decrease in network commonalities between phenotypes is a clear indicative that each one of the ccRC stages behave differently. This could be important, since each network maps a specific snapshot of the co-expression landscape at different moments of the carcinogenic process. Analysis of those unique co-expression features could help in the understanding of the cancer progression process.

#### 3.4.1. Most Interactions Are Phenotype-Specific

**Figure 7:**
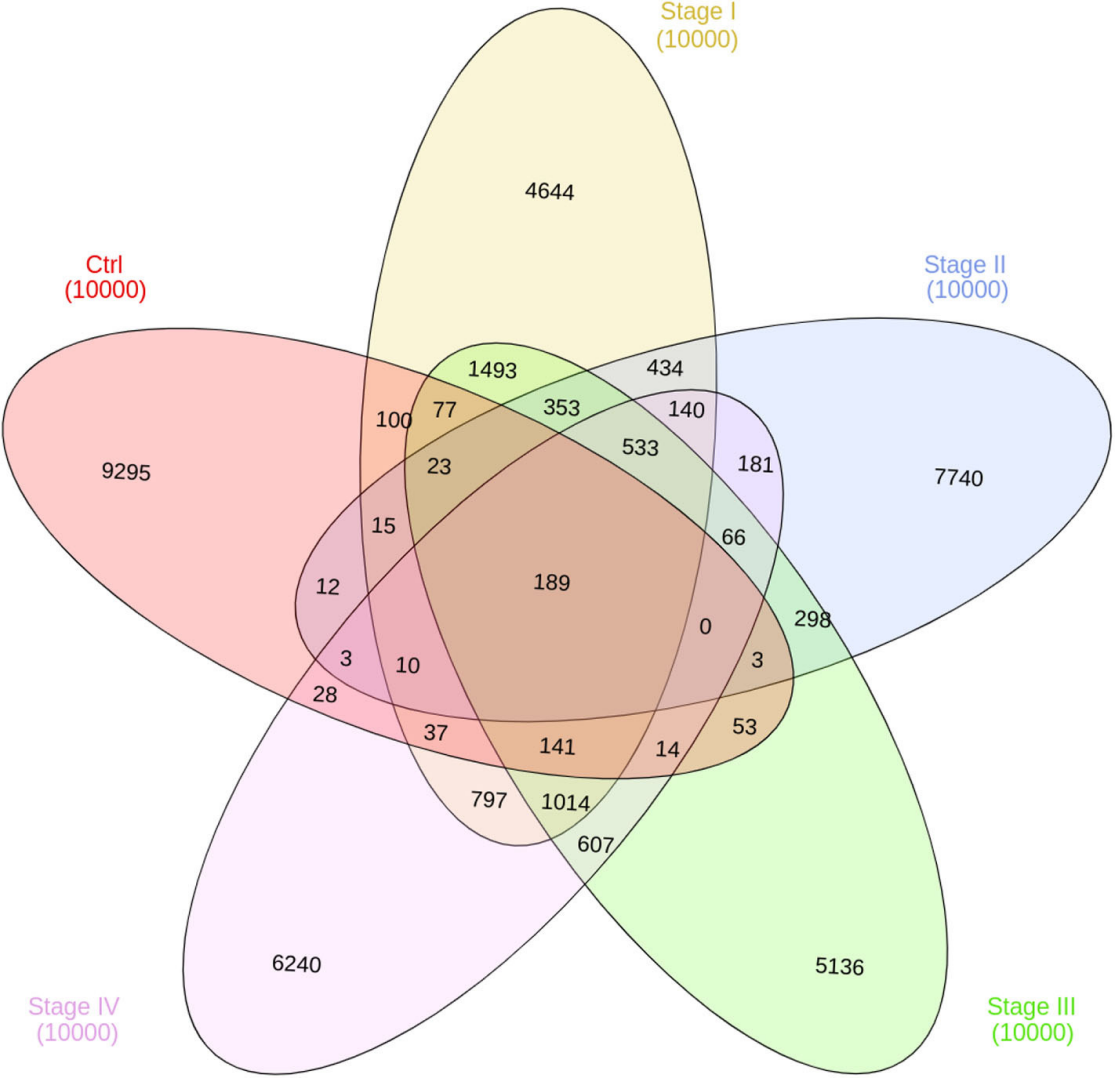
Edge intersection of all networks. Venn diagram shows, in each set, the number of edges per phenotype. The number reflect the shared genes between networks, as well as network-specific interactions. Notice that out of 10,000 interactions, only 189 edges are shared between the five networks.

In Figure 7, the intersection of co-expression interactions for the five phenotype networks is depicted. As it can be observed, the largest number of links belongs to the non-shared sets for the five networks. This indicates that, independently of the phenotype, networks are structurally different. As in the previous figure, the largest difference occurs in the control network (9,295 unique edges). Five thirty-three edges are shared between the four ccRC phenotypes. This is the set of co-expression interactions that appear at any stage of clear cell renal carcinoma.

### 3.5. Network Topologies at Different MI Cut-Offs

Since cut-off election is still a non-closed problem in network science (the so-called network sparsification problem), we decided to cover a wide range of cut-offs to assess the observed result in the previous sections. We pruned the original networks (16,000 genes, 130 millions of edges) into small mutual-information-ranked sets, from Top-100 to Top-1,000,000 edges, i.e., covering five orders of magnitude. See Supplementary Material 3.

#### 3.5.1. Proportion of Networks Intersection Decrease With Network Sizes

**Figure 8:**
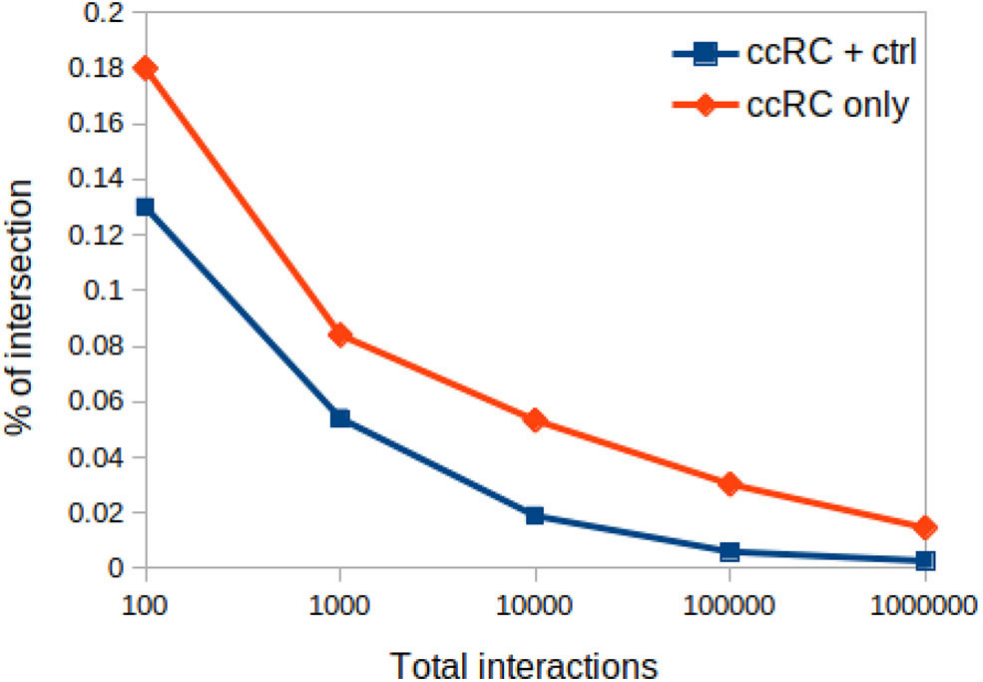
Proportion of networks intersection at different network cut-offs. In this plot, proportion of network intersection between the four ccRC stages (orange diamonds), and those with control network (blue squares) is depicted. X-axis represent different network cut-off values.

In Figure 8 one can appreciate that the proportion of intersections between all phenotypes (control and ccRC), as well as in ccRC-only networks, is maintained in a wide range of network cut-offs. It can be clearly appreciated the consecutive decrease of the proportion of shared links according to networks growing in size.

#### 3.5.2. Chromosomal Connectivity Differences Between Control and Cancer Networks Are Independent of the MI Cut-Off

**Figure 9:**
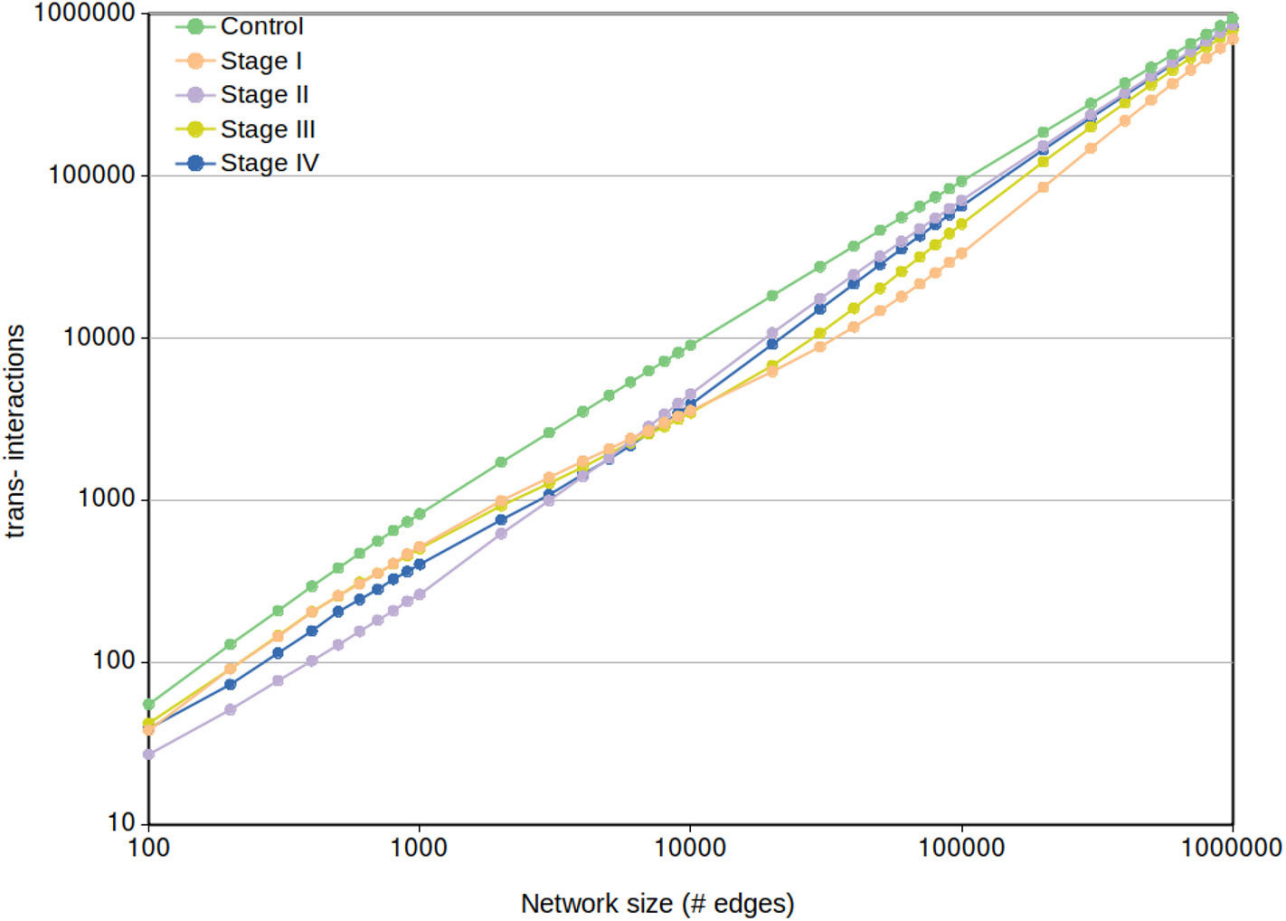
Network *trans-* interactions at different cut-offs. In this plot, X-axis represents the cut-off network value for the five different networks (control and the four ccRC stages). Y-axis shows the number of inter-chromosomal interactions per each network cut-off. To note that the control network *trans-* edges are larger than any ccRC progression stage at any cut-off network value.

Regarding the *cis-* and *trans-* difference between control and cancer networks, in Figure 9 we may observe that *trans-* interactions in control are always higher than any tumor ccRC network, despite the MI cut-off value.

It can be also appreciated that *trans-* interactions tend to converge according to the size increase. This result is expected since the more edges appear in the network, the more *cis-* edges have been “loaded” to prior cut-offs. This results also coincide with a recent finding in breast cancer networks, where consecutive non-overlapping layers of 100,000 edges (ranked top-to-bottom MI) contain more *cis-* interactions in top layers, and decreasing as they get close to the noise layer (Dorantes-Gilardi et al., 2020).

#### 3.5.3. Cancer Networks Present a Shift in the Order of cis-Rate in a Small Range of Interactions

In Figure 9 can also be observed that from the beginning range (100) to approximately 3,000 edges, the rank of *trans-* interactions is stage *I* → *III* → *IV* → *II*. However, in the range 3,000-to-10,000 edges this rank in ccRC networks changes from *I* → *III* → *IV* → *II* to *II* → *IV* → *III* → *I*. That acquired order is preserved until the already commented convergence at 1,000,000 edges.

As previously mentioned, the rank of *cis-/trans-* proportion does not follow progression of ccRC at any cut-off value. Hence we may conclude that differences in intra/inter-chromosomal network interactions are not a very informative parameter to evaluate progression in ccRC. Further investigation on the aforementioned shift is needed to have a more complete idea of the phenomenon.

### 3.6. 189 Biologically Relevant Edges Are Shared in the Five Phenotypes

**Figure 10:**
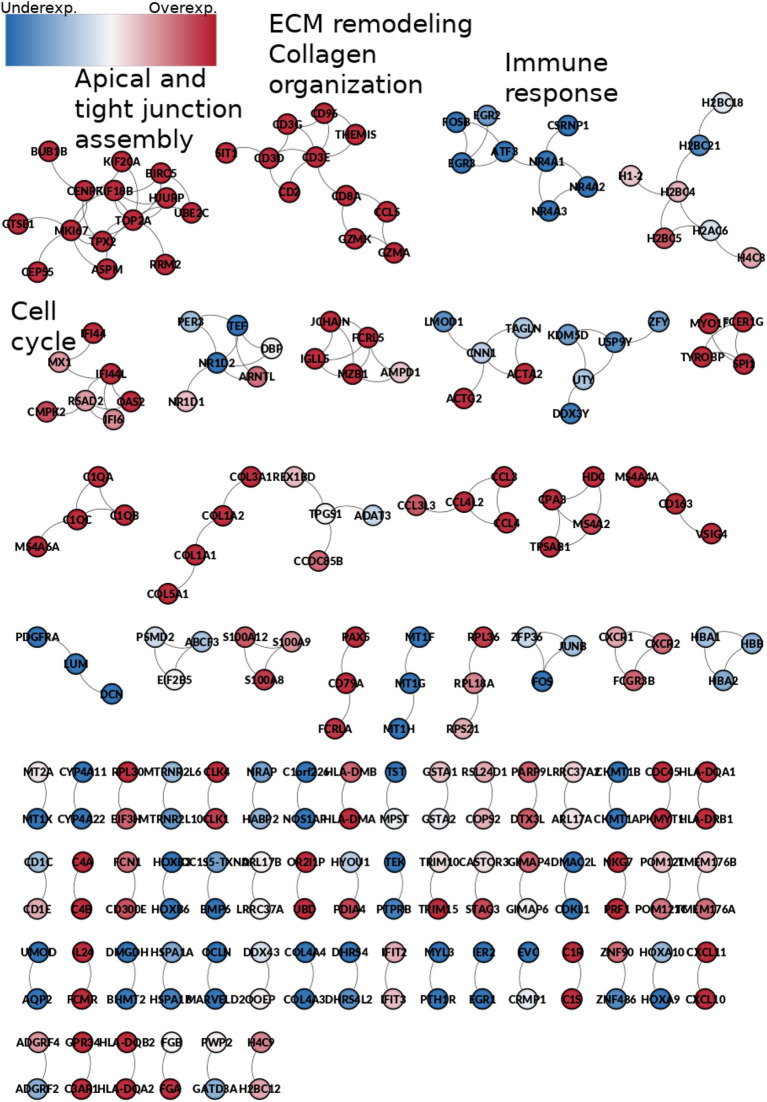
Network from shared interactions between the five phenotypes. The resulting network is composed of 189 edges and 230 genes. Those are colored according to the differential expression compared with the control group. Notice that network smaller components have a similar expression pattern. Some components are enriched to specific GO categories, meaning that those processes are increased or decreased during the whole process of ccRC progression.

189 co-expression interactions are shared between the five networks. Those interactions are depicted in Figure 10. The resulting network is composed of 230 genes and 189 edges. Genes are colored according to their differential gene expression.

Interestingly, network components of this common sub-network are mostly clustered according to the differential expression trend: there are clusters composed by over-expressed genes only, as well as under-expressed-only ones. It is worth mentioning that the Spearman's correlation between the rank of differentially expressed genes is higher than 0.95 for any stage (Table 2).

Additionally, the small connected components are enriched for particular and specific biological processes. For example, the first component, which contain genes such as KIF20A, KIF18B, or UBE2C, is enriched for apical and tight junction assembly. This is a highly overexpressed component, which indicates that for any stage, tight junction and apical junction assembly are exacerbated processes. Conversely, the third component, with genes such as EGR2, EGR3, ATF, or FOSB is completely underexpressed, and it is enriched for immune response-related processes, which could mean that the immune response is depleted at any stage of ccRC.

### 3.7. Enriched Categories Are Independent of the Cut-Off-Value

**Figure 11:**
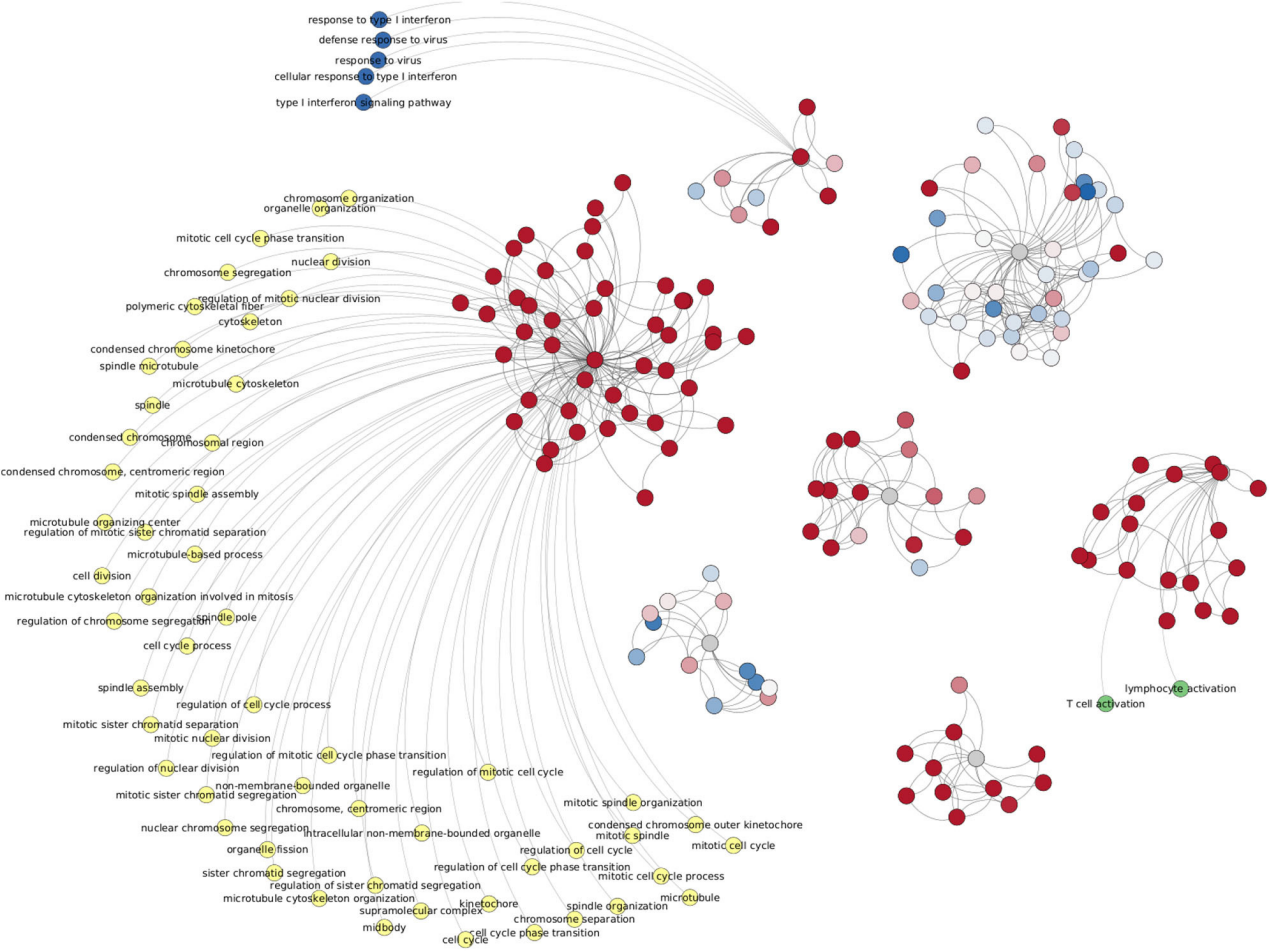
Network from shared interactions between Top-10,000 ccRC networks. The resulting network is composed of 533 edges and 148 genes. Those are colored according to their differential expression compared with the control group. As in case of Figure 10, differentially expressed clusters are enriched for specific categories.

Figure 11 shows the enriched categories obtained by intersecting the four progression stages (and excluding control interactions). Analog to Figure 10, in this case (533 edges) we have genes colored by their differential expression values, meanwhile enriched categories are painted by different colors depending on the component to which those processes belong. It is worth to mention that this figure only includes processes with a *p* − *value* < 10^−10^. The complete list of enriched processes for both cases, all phenotypes and ccRC-only, in the five cut-off network values, is included in Supplementary Material 4. Additionally, network visualizations of enriched processes in the ccRC-only network intersection for 100,000 and 1,000,000 interactions are also included in Supplementary Material 4.

Another shared feature of this figure with Figure 10 is that gene clusters with the same trend of differential expression have enriched categories. Among the top-enriched categories we may find cell-cycle-related (yellow), IFN-γ-related (dark blue), and T-cell-related (green) processes.

Since in this work one of the most relevant questions we made was related to the network structure at different progression stages in clear cell renal carcinoma, we calculated the over-expression analysis over network communities by means of the *infomap* algorithm (Rosvall and Bergstrom, 2008). We performed the enrichment analysis over separated sets of genes according to the community to which genes belong.

Given the fact that large networks often contains more communities than small networks, we performed the enrichment analysis for different cut-off values of network intersections. Independent of the network cut-off, intersections of ccRC-only networks always present this set of enriched categories, associated with cell-cycle, immune system, tridimensional structure of DNA and chromatin, or Transcription regulation (Supplementary Material 4).

## 4. Concluding Remarks

Clear cell renal carcinoma is a complex disease. It involves several layers of complexity. It must be dissected to have a comprehensive landscape allowing for a better understanding of its progression.

In previous work we observed an important increment in *cis*- ratio in breast cancer molecular subtypes, according to the malignancy of those phenotypes. Since the loss of long-range co-expression was observed in breast cancer and more remarkably in the Basal subtype (the one with worst prognosis), our working hypothesis was the more advanced the cancer stage, the higher the *cis-* ratio.

After breast cancer network analysis reported previously, clear cell renal carcinoma is the second cancer in which we observe a remarkable difference between *cis-* and *trans-* interactions, showing an important decrease in inter-chromosome gene-gene co-expression interactions in cancer networks.

Unexpectedly, the progression stage does not correlate with *cis-* ratio. This was observed not only in the top-10,000 edges networks, but also in a rank of five orders of magnitude. This could imply that *cis-* ratio is not a parameter to distinguish progression stages, at least for ccRC.

By observing the discrepancy between the *cis-* rate of ccRC progression stages with those observed in breast cancer molecular subtypes (García-Cortés et al., 2020), regarding that high proportion of intra-chromosome interactions are observed in those phenotypes with a worst prognosis we may argue the following:

- The fact that *cis-* rate does not coincide with progression stages, may reflect that high proportion of intra-chromosomal interactions are not a parameter to take into account to differentiate cancer progression, at least in clear cell renal carcinoma.
- A high *cis-* rate does not imply malignancy or worst prognosis in a cancerous network, but a different co-expression program in which gene-interactions are favored to physically close genes.
- The mechanisms behind the preferential co-expression to neighbor genes must imply epigenetic factors, such as micro-RNAs, lncRNAs, methylation profiles, tridimensional structure of DNA, chromatin modifications, CTCF binding sites, etc. (For a profound revision of spatial regulation of DNA in the oncogenic process, see Hernández-Lemus et al., 2019).

We want to stress that kidney cancers are fundamentally different from breast cancers in many forms (Hoadley et al., 2018). For the latter, topological similarities between breast cancer and ccRC co-expression networks must be taken carefully. However, it is remarkable that in both tissues (clear cell and breast carcinomas), as well as in separated instances (progression stages and molecular subtypes), the effect of loss of long-range co-expression is a common feature of cancer.

Here, we have focused on two main molecular signatures, namely the expression and the co-expression landscapes. In the first layer, we have observed that the differential expression profile is very similar between progression stages, even between stage I and stage IV, which may indicate that the expression profile is somehow acquired once cancer has started. However, certain genes appear to replicate the progression of oncogenic process, such as the case of SLC6A19 and PLG (underexpression), and SAAC2-SAAC4 and CXCL13 (overexpression). It is worth mentioning that none of these genes have been previously reported as progressively differentiated in renal carcinoma.

On the other hand, the similitude observed at the expression level, was not observed at the co-expression network level. Actually, the number of shared links is really low. We argue that the differential expression profiles are indeed insufficient to properly describe gene expression regulation, but the way that those genes interact in time and space is what ultimately determine the establishment of tumor phenotype.

In the case of Figure 6, the fact that chromosome Y is the only one with a higher *cis-* rate in control network than in ccRC stages may imply that, for this chromosome and its genes, local co-expression is crucial to maintain a proper functionality. It is widely known that two thirds of all ccRC cases correspond to men (Aron et al., 2008; Woldrich et al., 2008; Qu et al., 2015; Zaitsu et al., 2020). Since Chr Y is directly linked to gender, one may argue that an imbalance in the *cis-/trans-* proportion may be implicated in gender-bias on clear cell renal carcinoma.

Despite the high differences between control and stage networks, and even between stages, there are some conserved gene co-expression relationships independent of the phenotype. An instance of this is shown Figure 10. Those interactions shared among the five phenotypes show very few common links, but clustered in biologically relevant genesets. Those genesets are important for cell maintenance (that is perhaps, the reason for which they appear in the control network). At the same time, these genesets are overexpressed, thus indicating an exacerbated process in the cancer stages, as in the case of apical and tight junction assembly, or extracellular matrix remodeling.

Conversely, the immune response cluster is depleted, thus indicating that the immune system response may be decreased at any moment in the course of the carcinogenic process.

Analogously, in Figure 11 we may observe the shared interactions between cancer-only networks. This subset of interactions may result of the utmost relevance, since it represents those gene-gene co-expression relationships that are exclusive of clear cell renal carcinoma. These interactions are highly enriched for very specific biological processes, which means that these interactions may have repercussion in cell functionality. Another point to remark regarding ccRC-only intersection is that the enriched functions are preserved at five orders of magnitude network sizes.

The fact that topological and functional analyses show similar results at five orders of magnitude in network sizes, have implications in at least two main issues: (a) *cis-* rate is invariant to the cut-off, and (b) enriched categories do not depend of the cut-off value. Here, we have provided a methodology to discover functional characteristics of gene-co-expression networks that are intrinsic to the phenotype and not depend on the network cut-off.

We are aware that gene co-expression may be strongly influenced by several factors: micro-RNAs, long non-coding RNAs, methylation patterns, copy number alterations, 3D-structure of DNA, CTCFs binding sites, to mention but a few. More research is thus needed for a better understanding of the delicate interplay between gene expression and co-expression. This is a first approach to draw close both worlds in an integrative manner.

## Data Availability Statement

The datasets presented in this study can be found in online repositories. The names of the repository/repositories and accession number(s) can be found in the article/Supplementary Material.

## Author Contributions

JZ-F performed computational analyses, developed and implemented programming code, performed pre-processing and low-level data analysis, made the figures, and drafted the manuscript. EH-L contributed to design the theoretical and modeling analysis and contributed and supervised the writing of the manuscript. JE-E conceived and designed the project, supervised the project, performed biological analyses, and drafted the manuscript. All authors read and approved the final version of the manuscript.

## Conflict of Interest

The authors declare that the research was conducted in the absence of any commercial or financial relationships that could be construed as a potential conflict of interest.

## Supplementary Material

Supplementary Material 1 Quality control for gene expression in the five phenotypes. This zip file contains five folders with the quality control pre and post normalization of gene expression data. They include length bias correction, GC-content correction, and PCA for the five groups.

Supplementary Material 2 Differential expression values for each ccRC stage vs. control samples. This zip file contains the four differential expression analyses, including *Log*_2_*FC* value, *p*-value, adjusted *p*-value, and B-statistic for each gene in all stages. HTML files for volcano plots are also provided.

Supplementary Material 3 Heatmaps for intersections and differences in all phenotypes with MI cut-offs of 100, 1,000, 10,000, 100,000, and 1,000,000 interactions. Venn diagrams for intersections of all phenotypes with the aforementioned cut-off values.

Supplementary Material 4 Complete list of enriched processes for network intersections at different cut-off values. These files contain the enriched categories for both sets, all-phenotypes (control and the four progression stages), as well as ccRC-only intersections. In all cases, the list of enriched categories was performed over network communities, not over the whole geneset. Files are separated based on the cut-off of networks. Visualization of enriched process in Networks of 100,000 and 1,000,000 MI cuts.

## Abbreviations

ccRC: Clear Cell Renal Carcinoma
Log_2_FC: Log_2_Fold Change
MI: Mutual Information
TCGA: The Cancer Genome Atlas.
