# Supplementary figures and images for: Gene Expression and Co-expression Networks Are Strongly Altered Through Stages in Clear Cell Renal Carcinoma
Source: Front Genet. 2020 Nov 3;11:578679. doi: 10.3389/fgene.2020.578679 (PMC7669746; doi:10.3389/fgene.2020.578679)

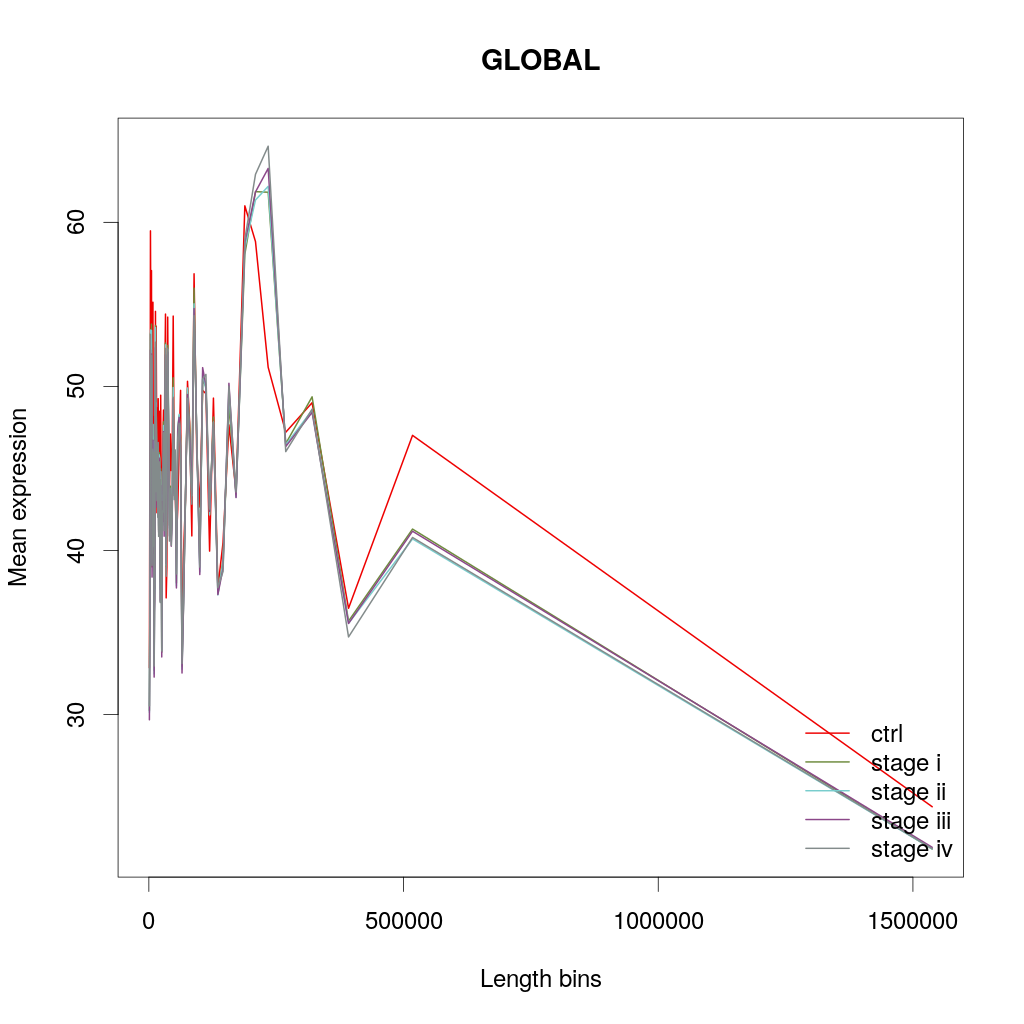

Supplement: Supplementary Material 1 — Quality control for gene expression in the five phenotypes. This zip file contains five folders with the quality control pre and post normalization of gene expression data. They include length bias correction, GC-content correction, and PCA for the five groups. [file Data_Sheet_1.ZIP › QC_POST/01-Lengthbias.png]

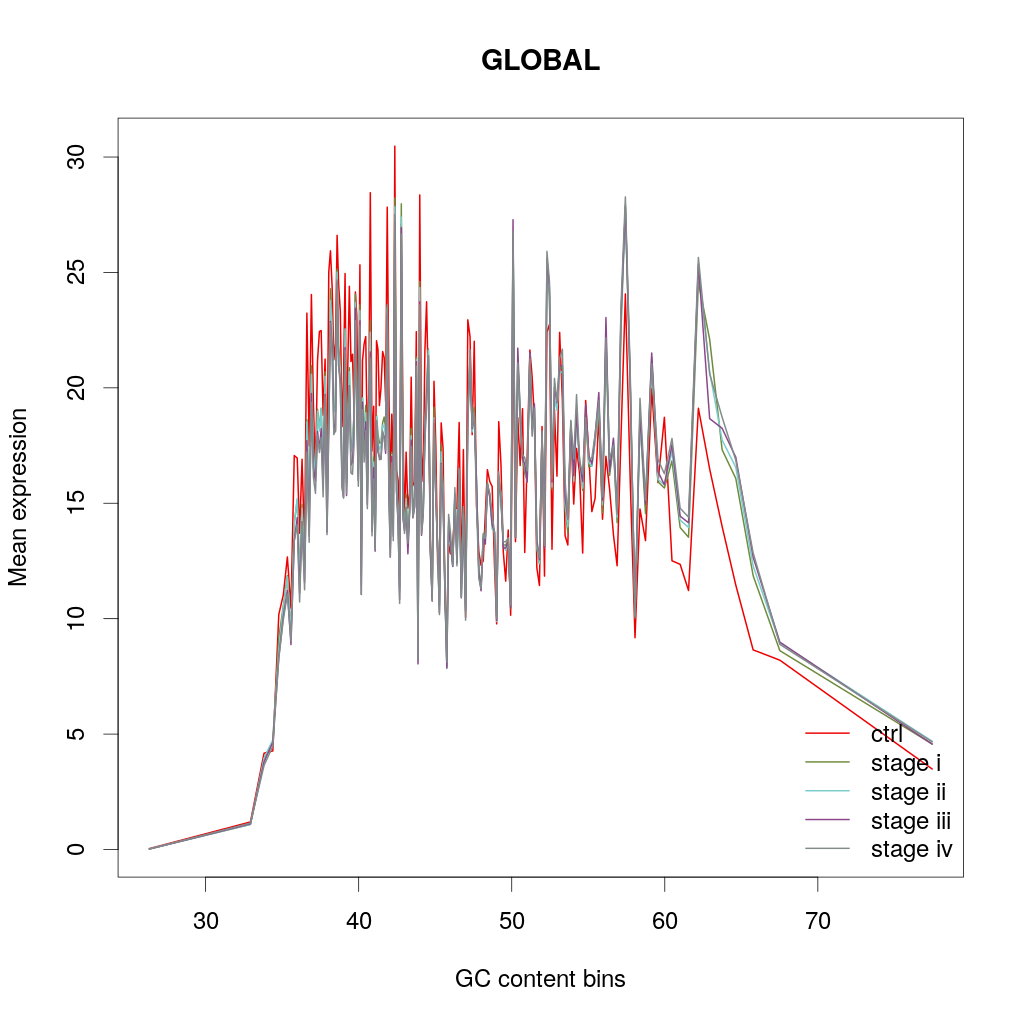

Supplement: Supplementary Material 1 — Quality control for gene expression in the five phenotypes. This zip file contains five folders with the quality control pre and post normalization of gene expression data. They include length bias correction, GC-content correction, and PCA for the five groups. [file Data_Sheet_1.ZIP › QC_POST/02-GCbias.png]

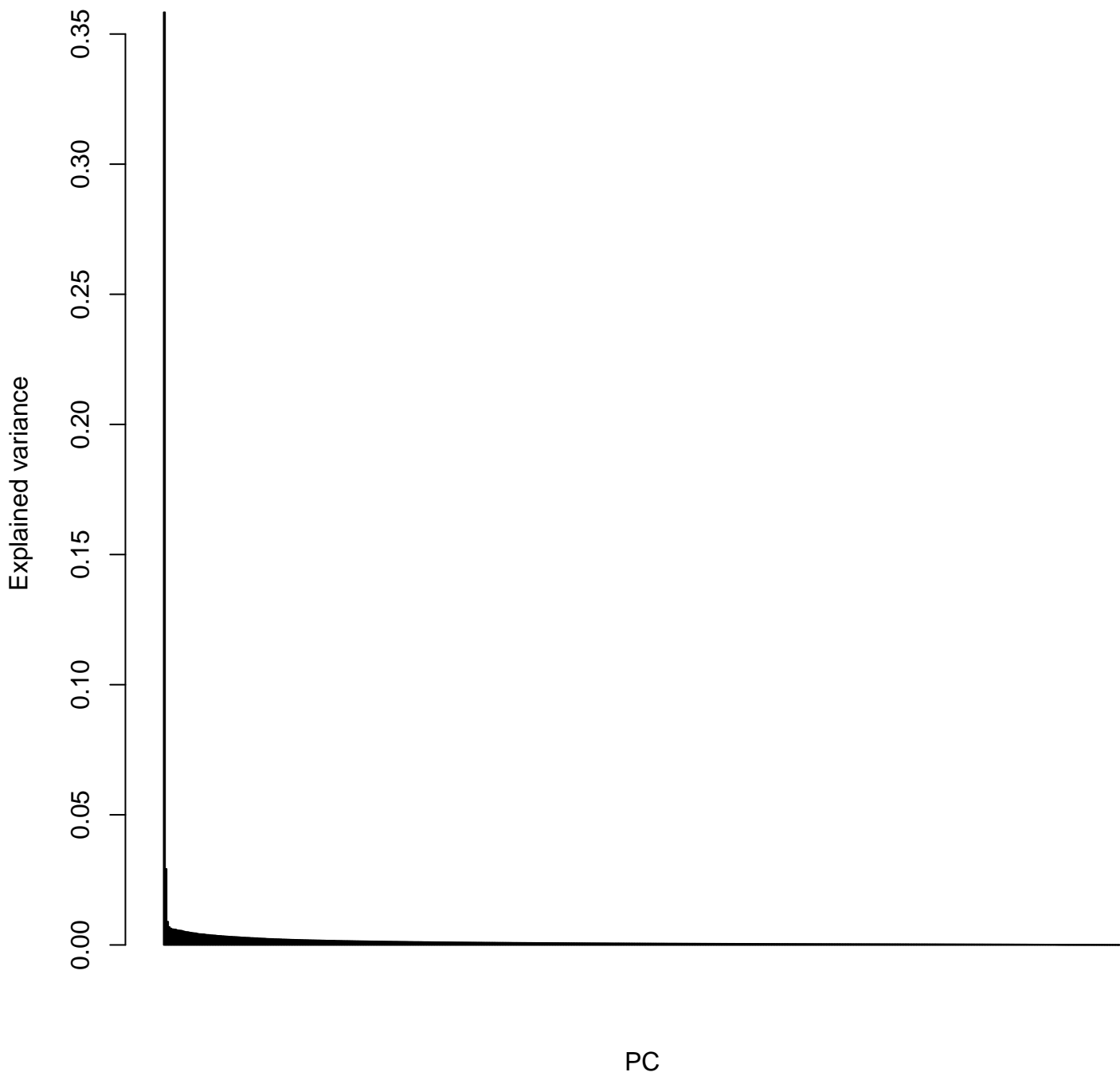

Supplement: Supplementary Material 1 — Quality control for gene expression in the five phenotypes. This zip file contains five folders with the quality control pre and post normalization of gene expression data. They include length bias correction, GC-content correction, and PCA for the five groups. [file Data_Sheet_1.ZIP › QC_POST/03-PCAVariance_raw.pdf]

PCA loadings

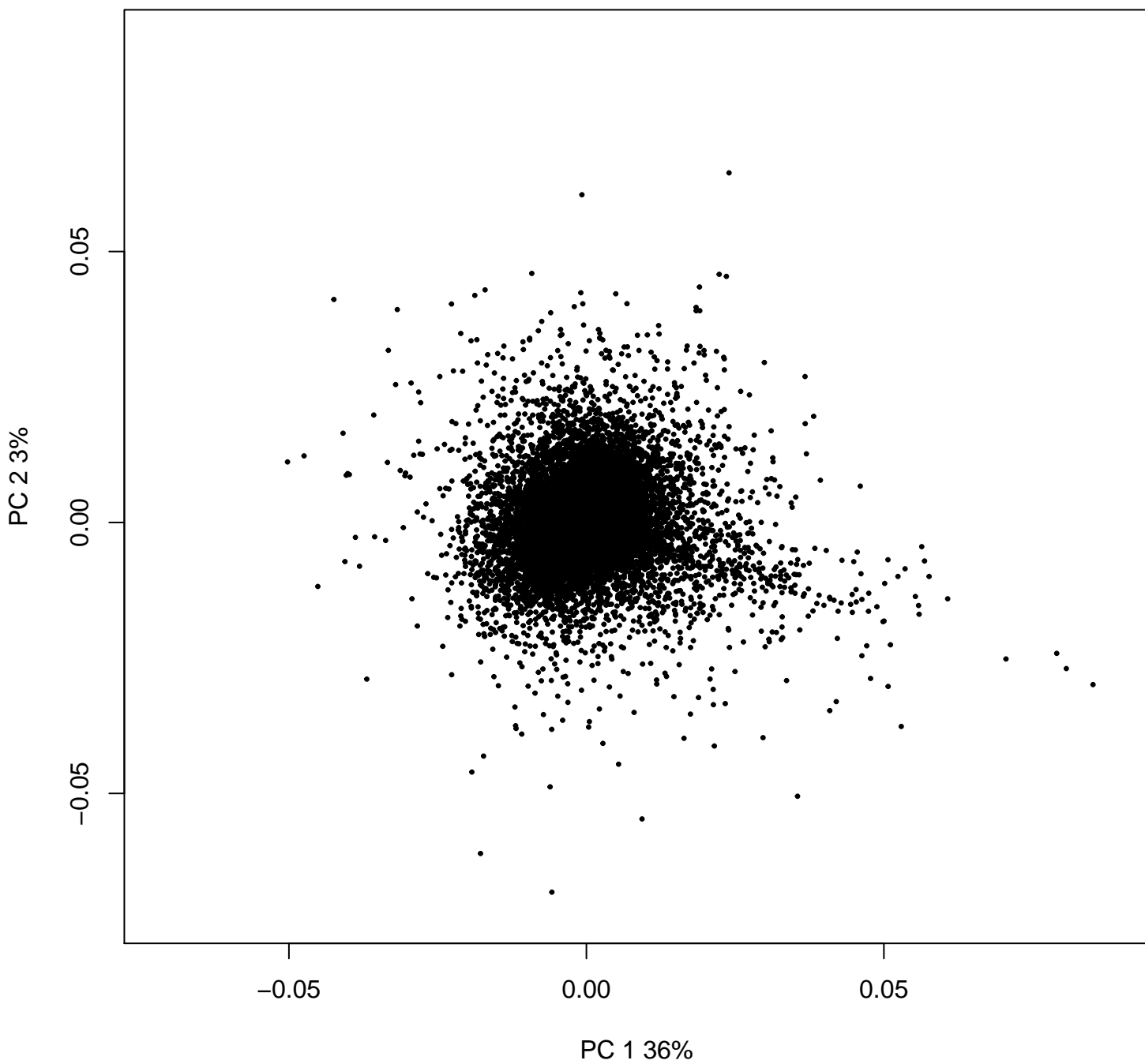

Supplement: Supplementary Material 1 — Quality control for gene expression in the five phenotypes. This zip file contains five folders with the quality control pre and post normalization of gene expression data. They include length bias correction, GC-content correction, and PCA for the five groups. [file Data_Sheet_1.ZIP › QC_POST/04-PCALoading_raw.pdf]

PCA scores

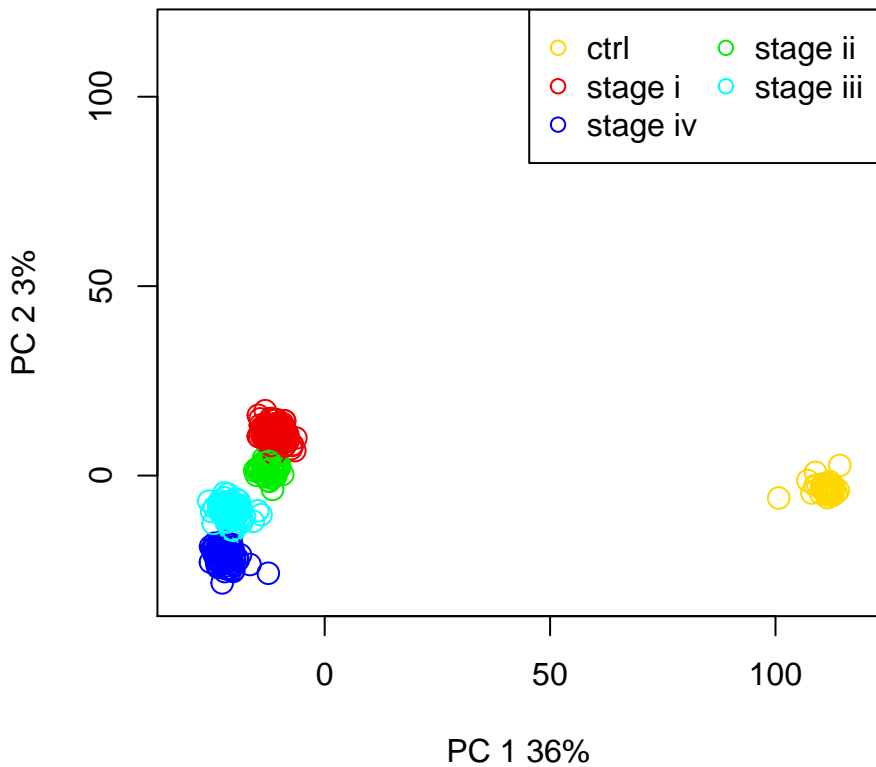

PCA scores

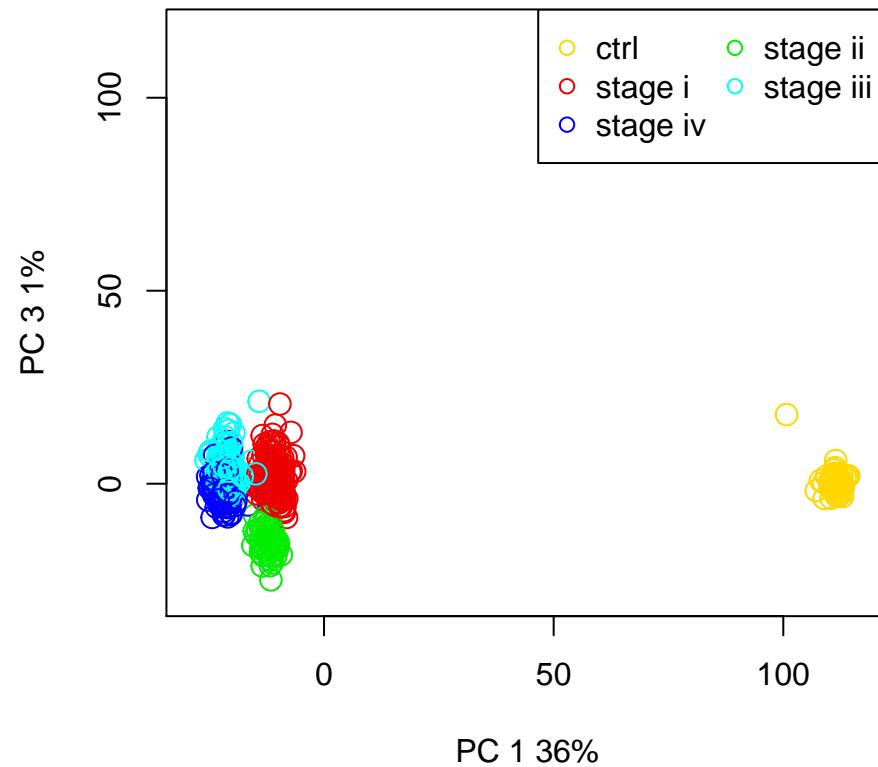

Supplement: Supplementary Material 1 — Quality control for gene expression in the five phenotypes. This zip file contains five folders with the quality control pre and post normalization of gene expression data. They include length bias correction, GC-content correction, and PCA for the five groups. [file Data_Sheet_1.ZIP › QC_POST/05-PCAScore_raw.pdf]

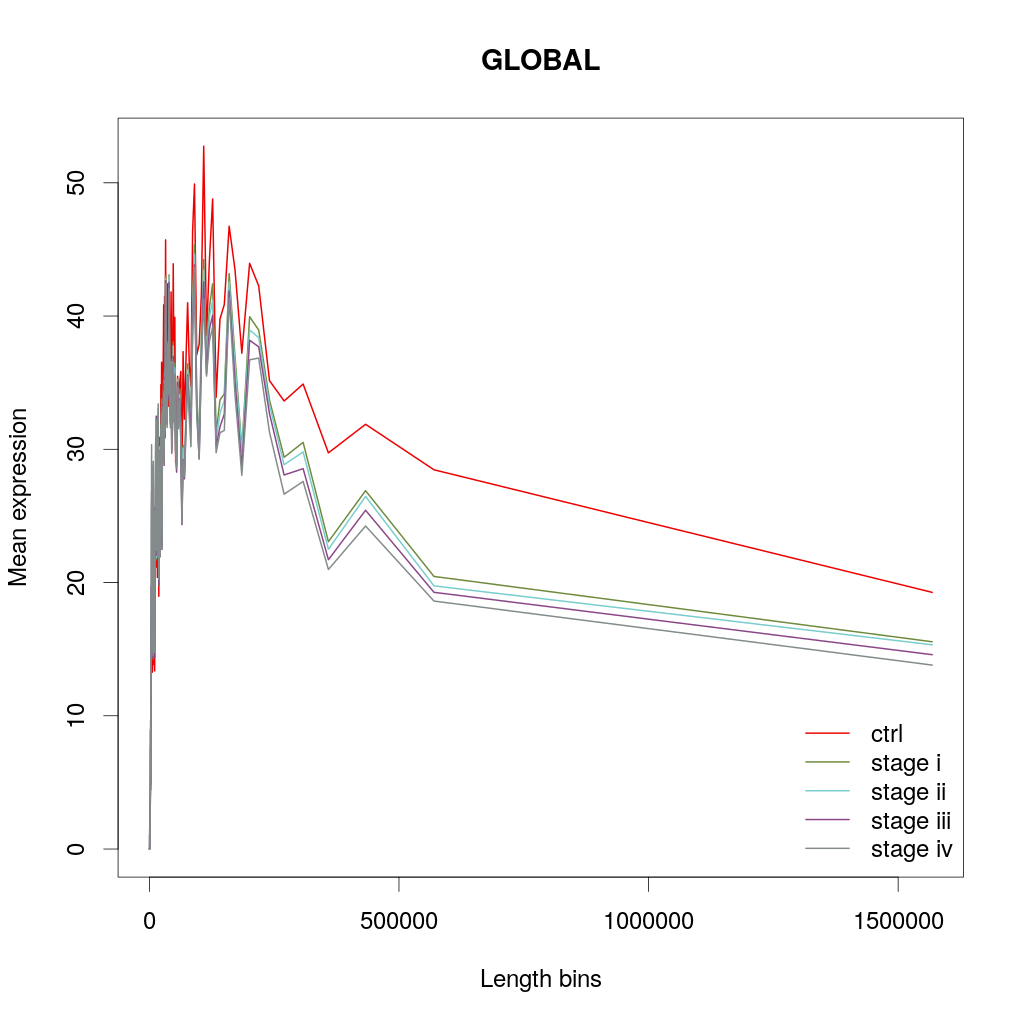

Supplement: Supplementary Material 1 — Quality control for gene expression in the five phenotypes. This zip file contains five folders with the quality control pre and post normalization of gene expression data. They include length bias correction, GC-content correction, and PCA for the five groups. [file Data_Sheet_1.ZIP › QC_PRE/05-Lengthbias.png]

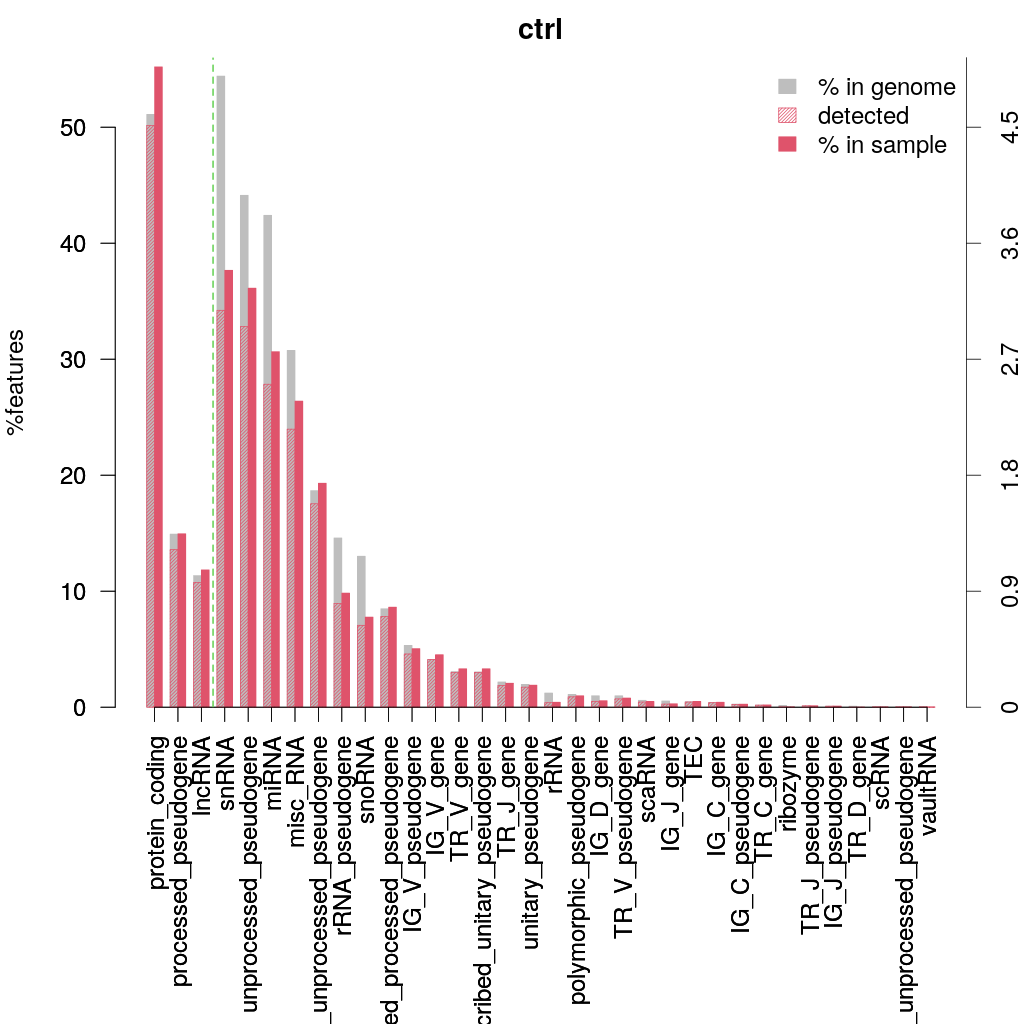

Supplement: Supplementary Material 1 — Quality control for gene expression in the five phenotypes. This zip file contains five folders with the quality control pre and post normalization of gene expression data. They include length bias correction, GC-content correction, and PCA for the five groups. [file Data_Sheet_1.ZIP › QC_PRE/01-biodetection.Rd_00001.png]

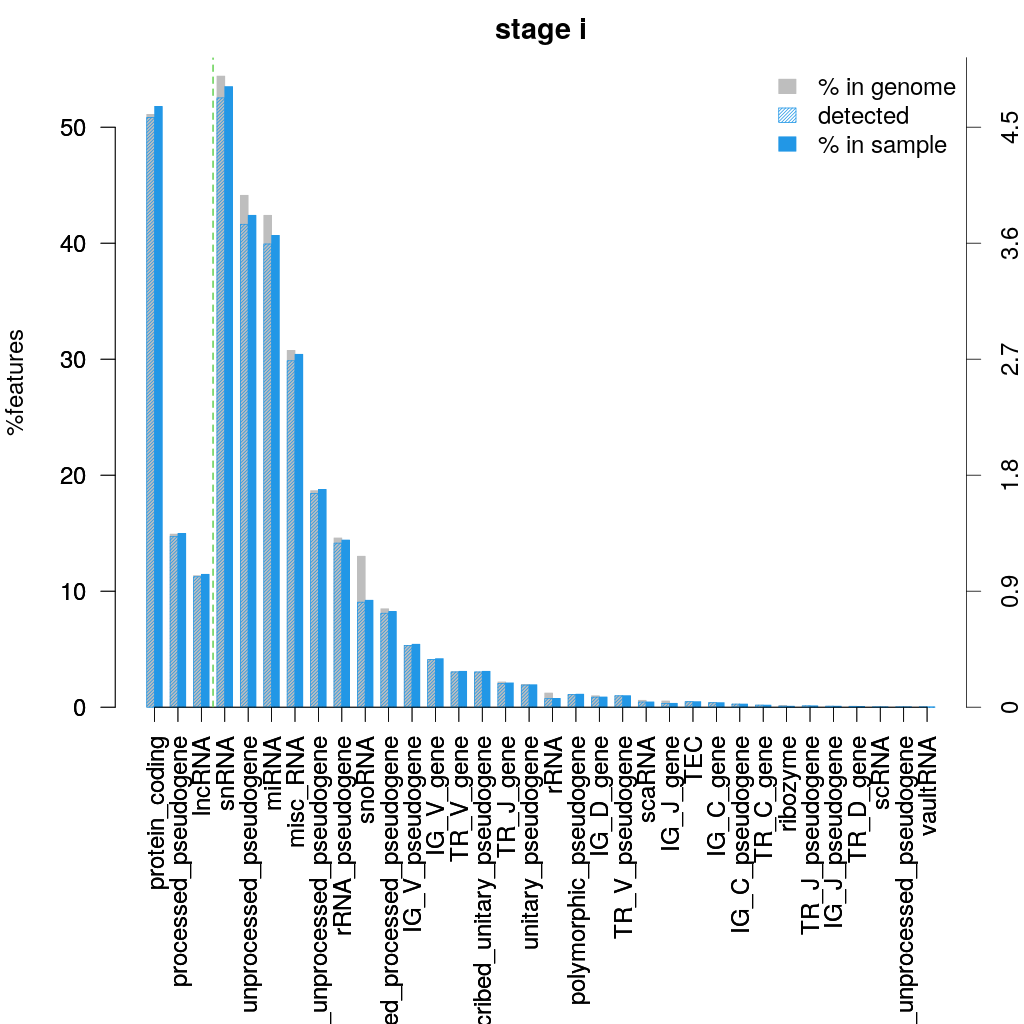

Supplement: Supplementary Material 1 — Quality control for gene expression in the five phenotypes. This zip file contains five folders with the quality control pre and post normalization of gene expression data. They include length bias correction, GC-content correction, and PCA for the five groups. [file Data_Sheet_1.ZIP › QC_PRE/01-biodetection.Rd_00002.png]

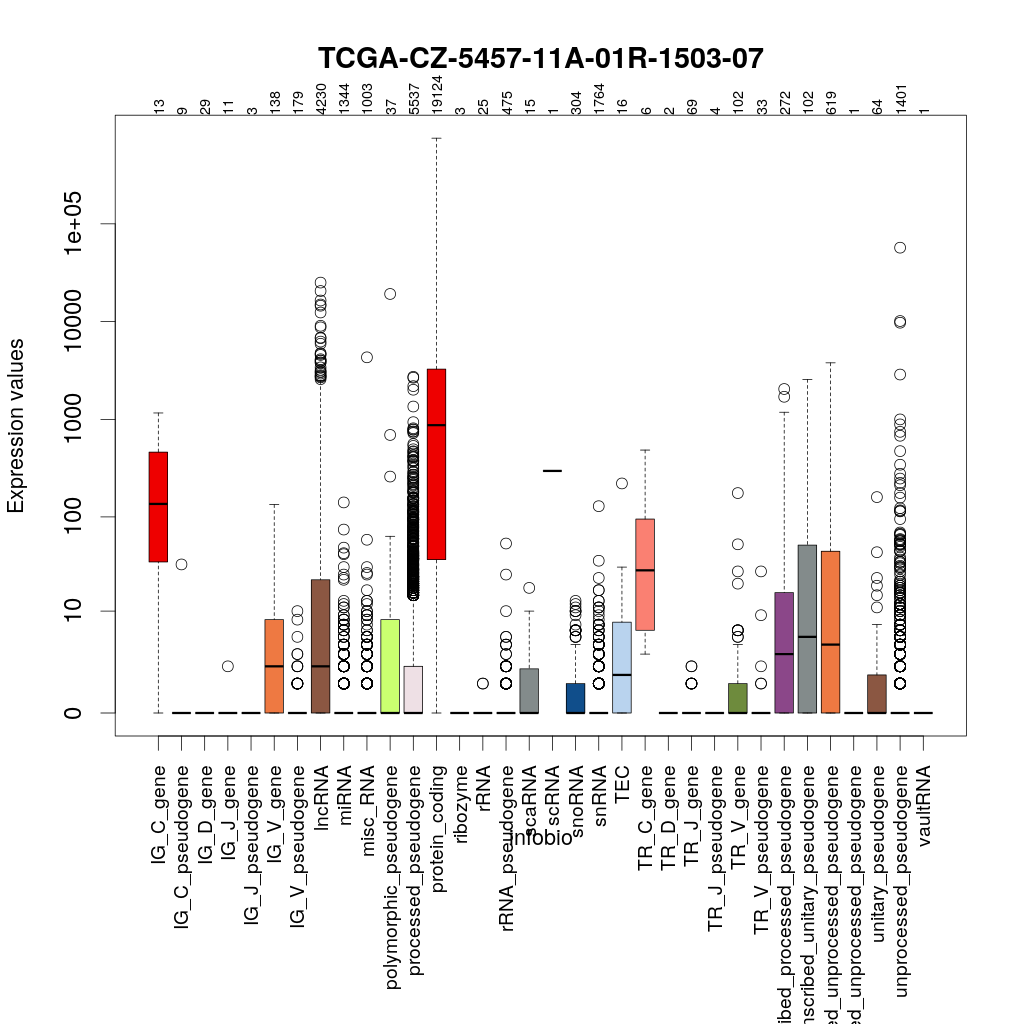

Supplement: Supplementary Material 1 — Quality control for gene expression in the five phenotypes. This zip file contains five folders with the quality control pre and post normalization of gene expression data. They include length bias correction, GC-content correction, and PCA for the five groups. [file Data_Sheet_1.ZIP › QC_PRE/02-countsbio.png]

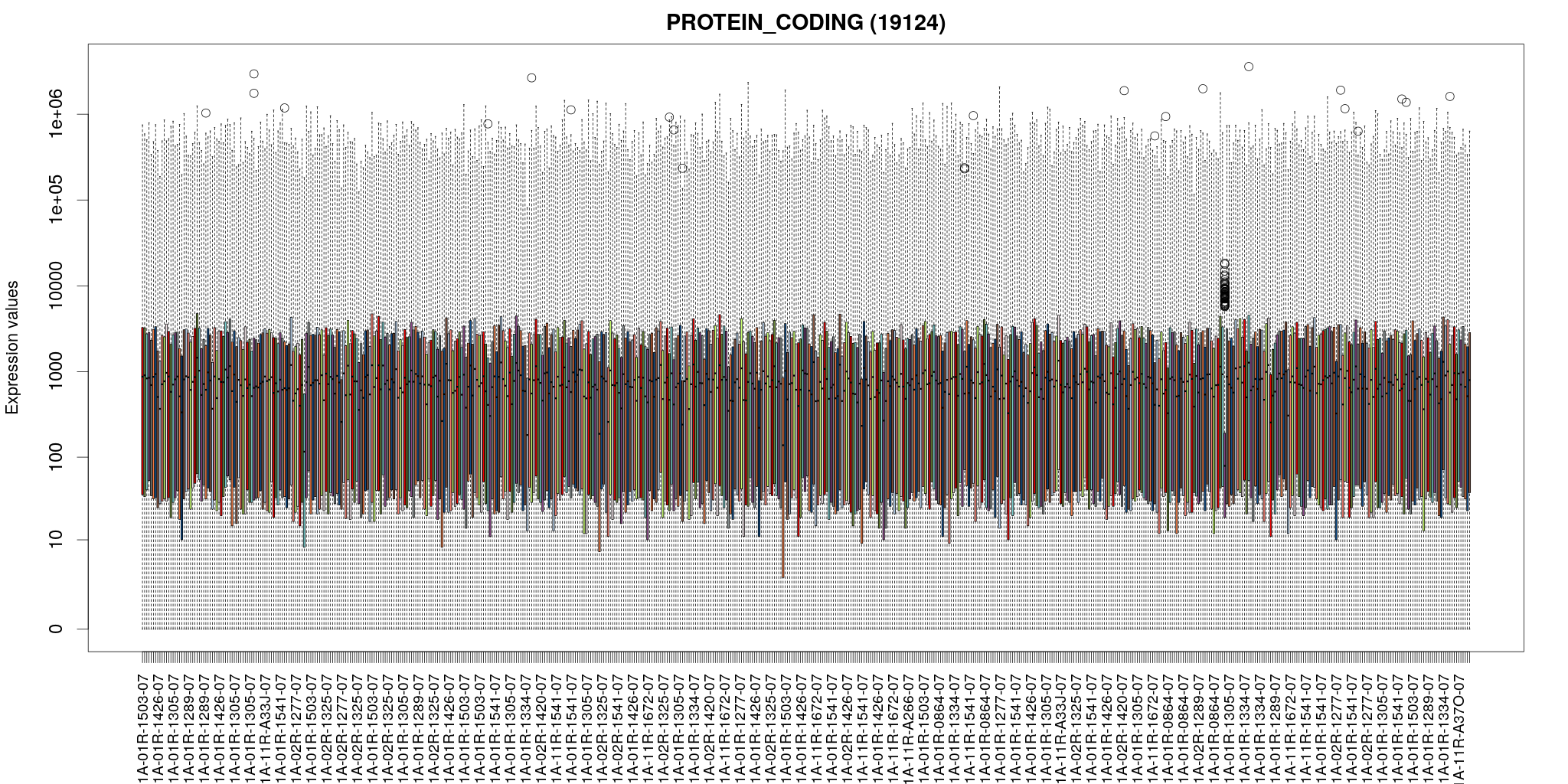

Supplement: Supplementary Material 1 — Quality control for gene expression in the five phenotypes. This zip file contains five folders with the quality control pre and post normalization of gene expression data. They include length bias correction, GC-content correction, and PCA for the five groups. [file Data_Sheet_1.ZIP › QC_PRE/02-protein_coding_boxplot.png]

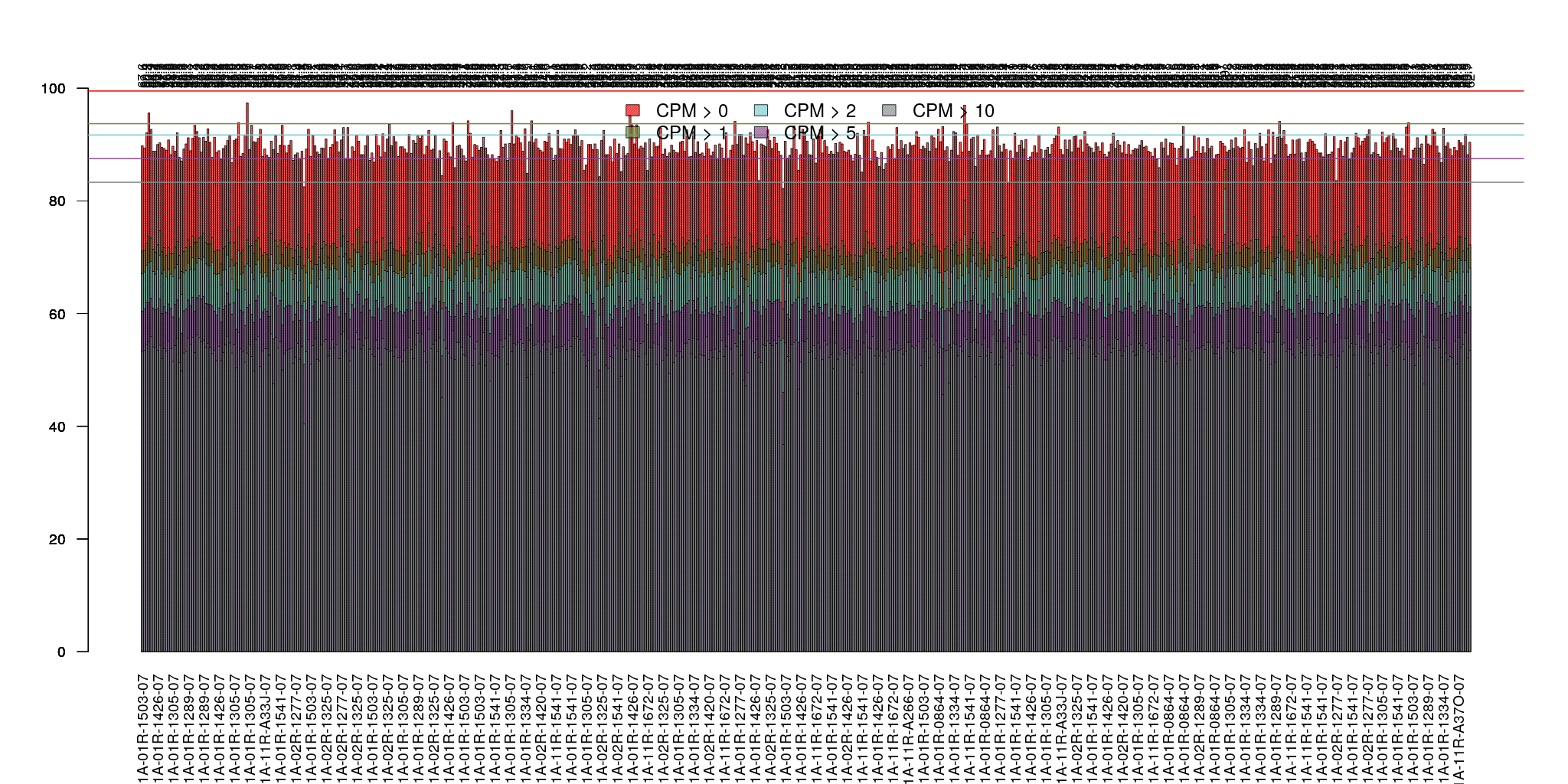

Supplement: Supplementary Material 1 — Quality control for gene expression in the five phenotypes. This zip file contains five folders with the quality control pre and post normalization of gene expression data. They include length bias correction, GC-content correction, and PCA for the five groups. [file Data_Sheet_1.ZIP › QC_PRE/02-protein_coding_barplot.png]

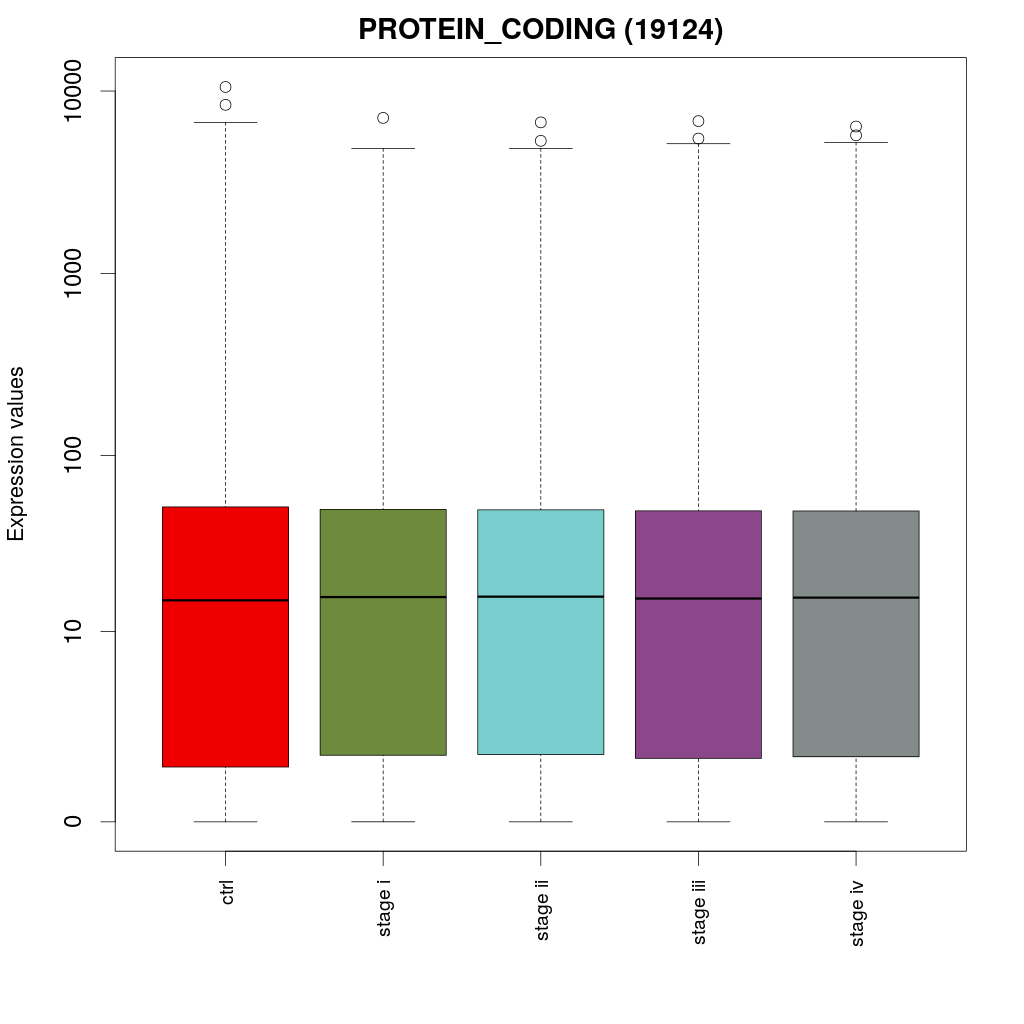

Supplement: Supplementary Material 1 — Quality control for gene expression in the five phenotypes. This zip file contains five folders with the quality control pre and post normalization of gene expression data. They include length bias correction, GC-content correction, and PCA for the five groups. [file Data_Sheet_1.ZIP › QC_PRE/03-protein_coding_boxplot_group.png]

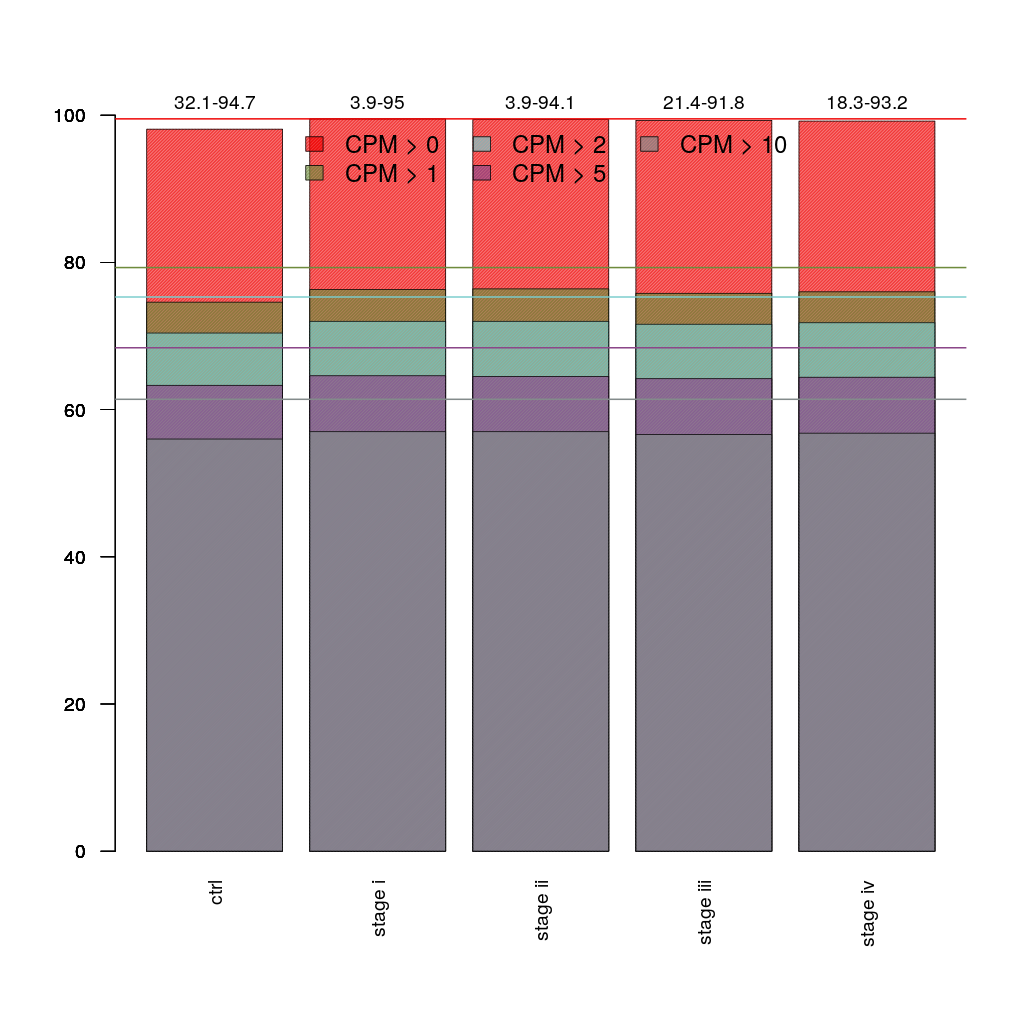

Supplement: Supplementary Material 1 — Quality control for gene expression in the five phenotypes. This zip file contains five folders with the quality control pre and post normalization of gene expression data. They include length bias correction, GC-content correction, and PCA for the five groups. [file Data_Sheet_1.ZIP › QC_PRE/04-protein_coding_barplot_group.png]

Explained variance

0.15

0.10

0.05

0.00

PC

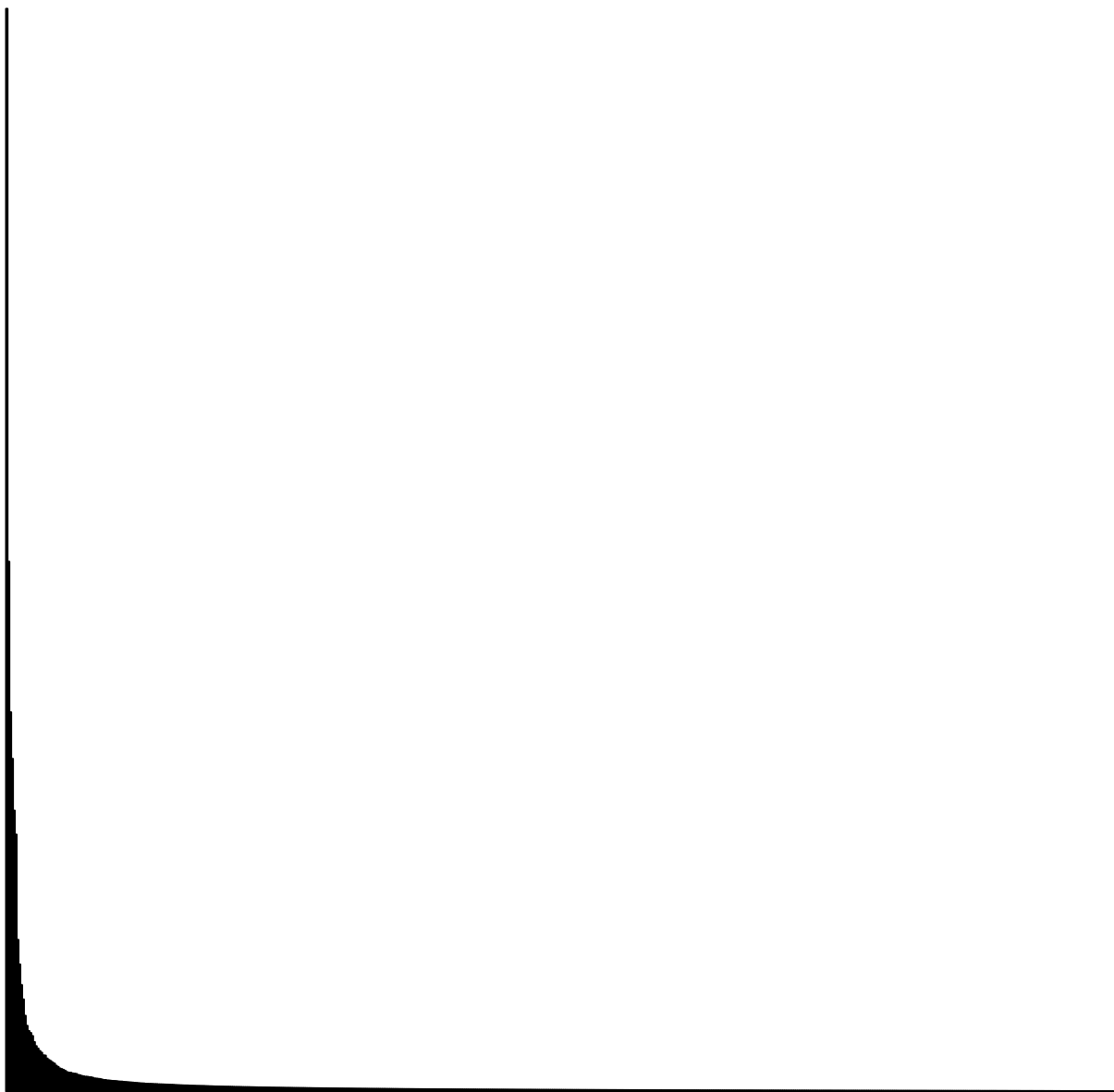

Supplement: Supplementary Material 1 — Quality control for gene expression in the five phenotypes. This zip file contains five folders with the quality control pre and post normalization of gene expression data. They include length bias correction, GC-content correction, and PCA for the five groups. [file Data_Sheet_1.ZIP › QC_PRE/08-PCAVariance_raw.pdf]

# PCA loadings

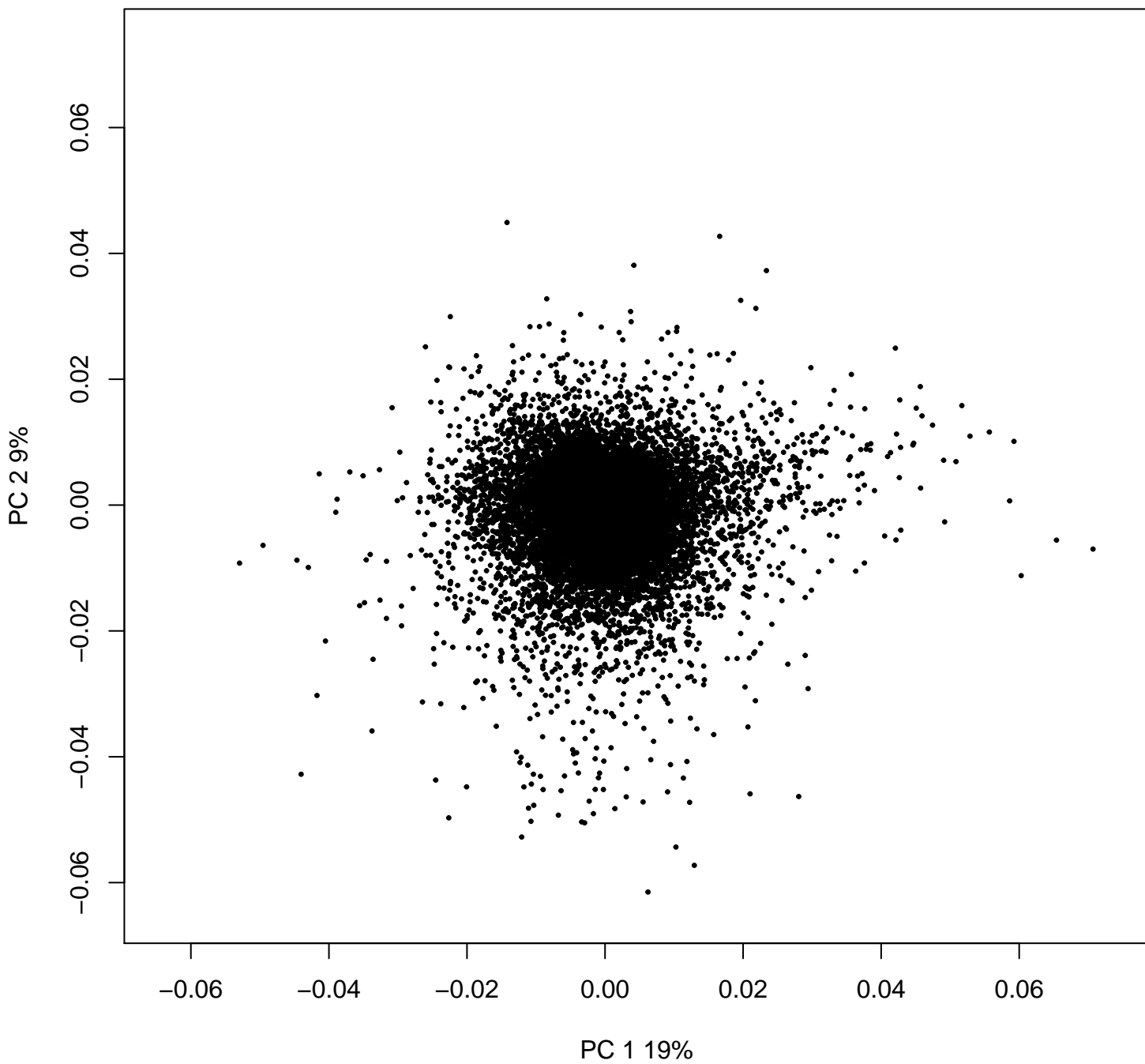

Supplement: Supplementary Material 1 — Quality control for gene expression in the five phenotypes. This zip file contains five folders with the quality control pre and post normalization of gene expression data. They include length bias correction, GC-content correction, and PCA for the five groups. [file Data_Sheet_1.ZIP › QC_PRE/09-PCALoading_raw.pdf]

**PCA scores**

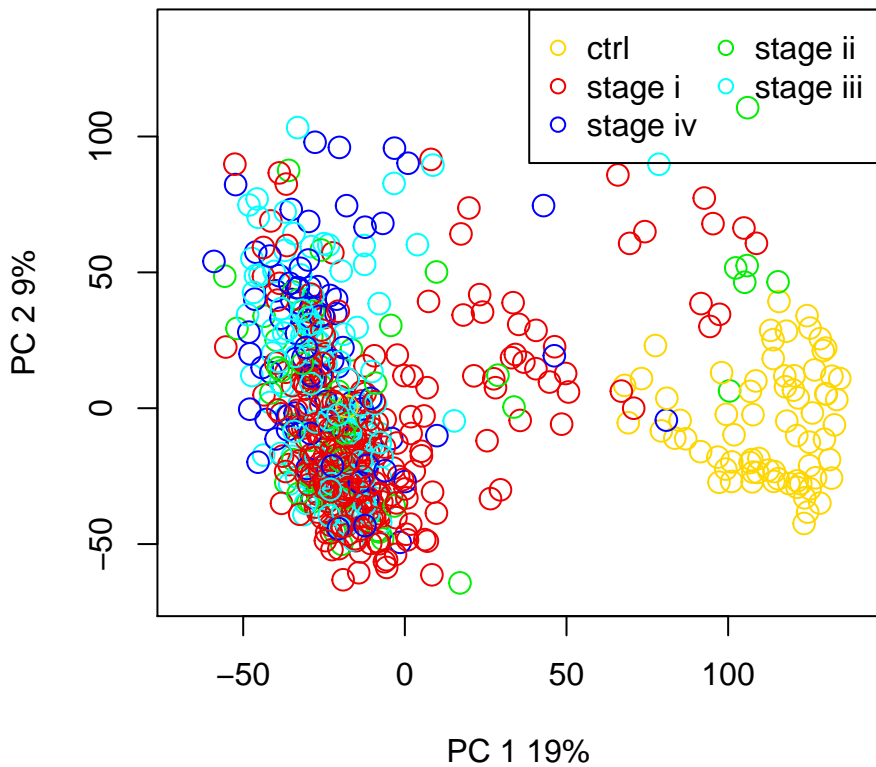

**PCA scores**

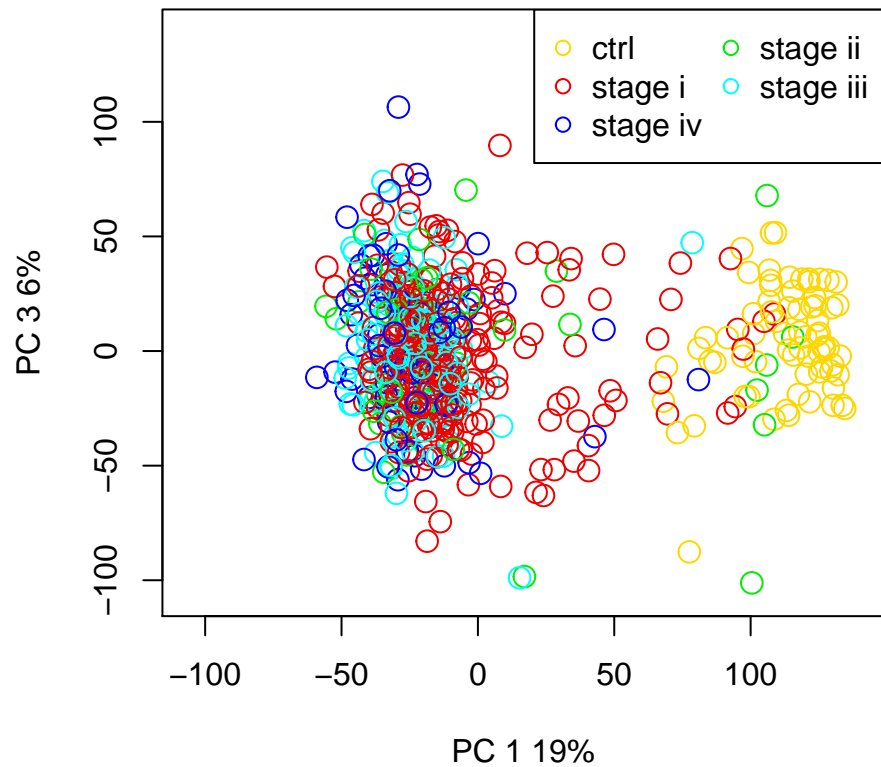

Supplement: Supplementary Material 1 — Quality control for gene expression in the five phenotypes. This zip file contains five folders with the quality control pre and post normalization of gene expression data. They include length bias correction, GC-content correction, and PCA for the five groups. [file Data_Sheet_1.ZIP › QC_PRE/10-PCAScore_raw.pdf]

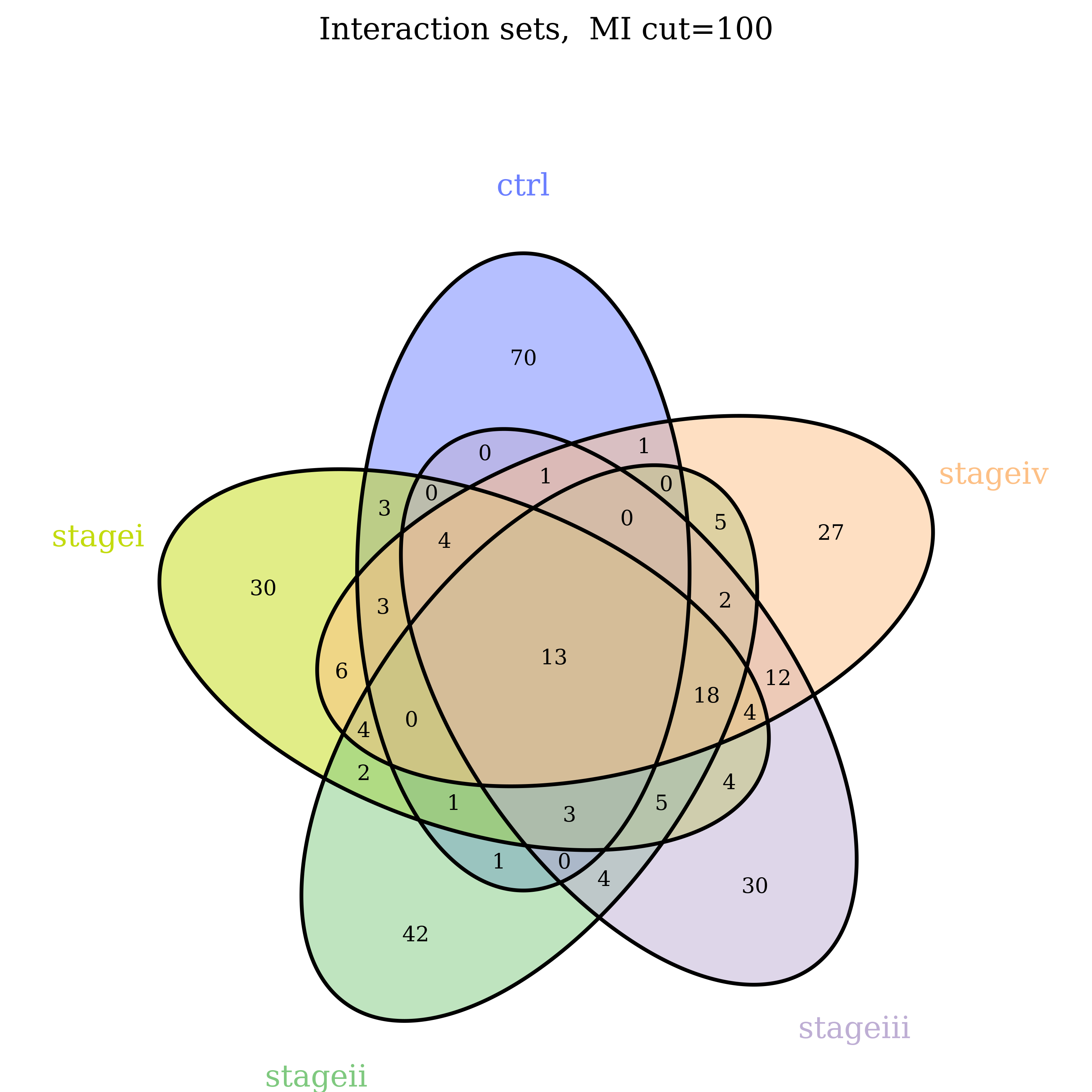

Supplement: Supplementary Material 3 — Heatmaps for intersections and differences in all phenotypes with MI cut-offs of 100, 1,000, 10,000, 100,000, and 1,000,000 interactions. Venn diagrams for intersections of all phenotypes with the aforementioned cut-off values. [file Data_Sheet_3.ZIP › Supp_Mat_3/Venn/venn-100.png]

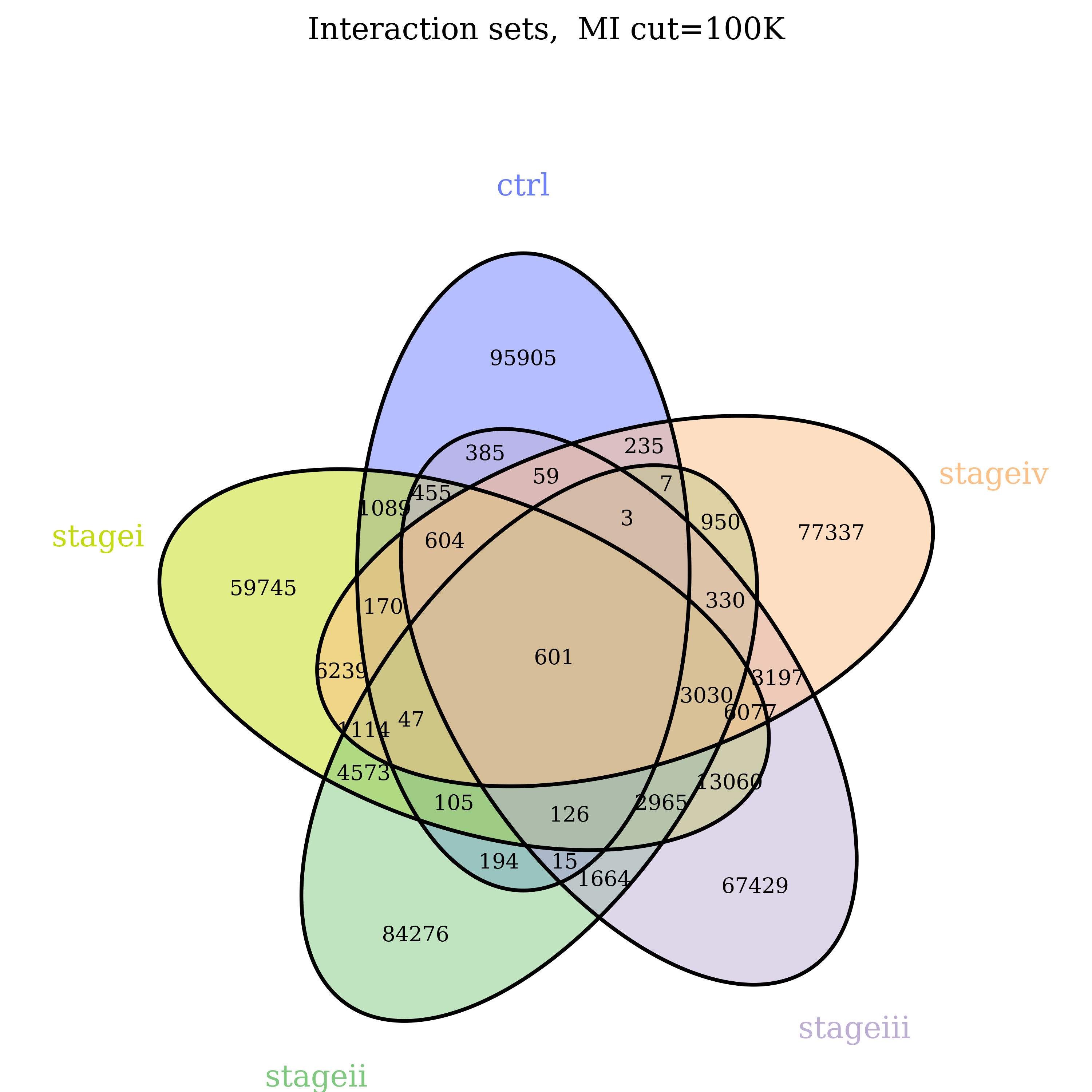

Supplement: Supplementary Material 3 — Heatmaps for intersections and differences in all phenotypes with MI cut-offs of 100, 1,000, 10,000, 100,000, and 1,000,000 interactions. Venn diagrams for intersections of all phenotypes with the aforementioned cut-off values. [file Data_Sheet_3.ZIP › Supp_Mat_3/Venn/venn-100K.png]

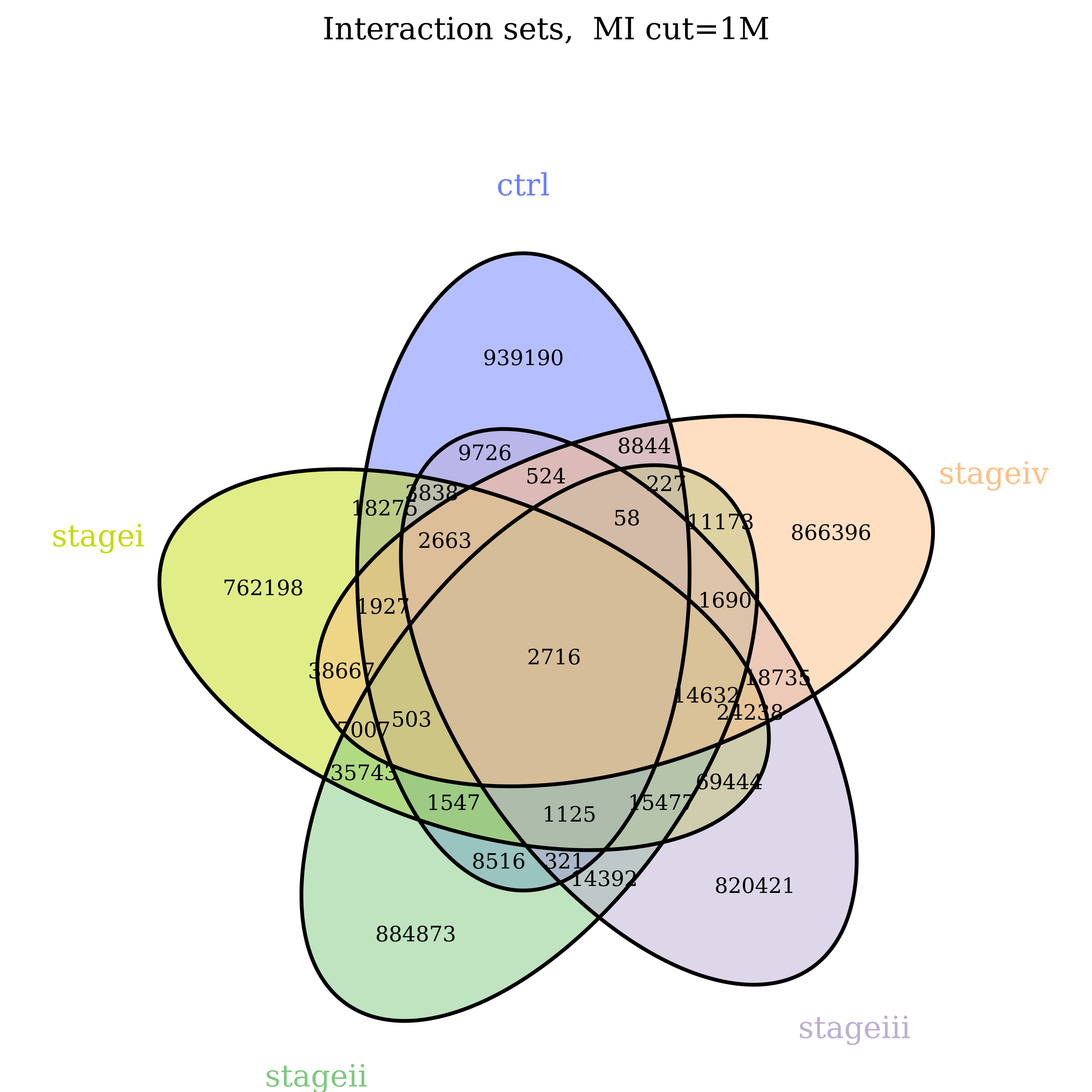

Supplement: Supplementary Material 3 — Heatmaps for intersections and differences in all phenotypes with MI cut-offs of 100, 1,000, 10,000, 100,000, and 1,000,000 interactions. Venn diagrams for intersections of all phenotypes with the aforementioned cut-off values. [file Data_Sheet_3.ZIP › Supp_Mat_3/Venn/venn-1M.png]

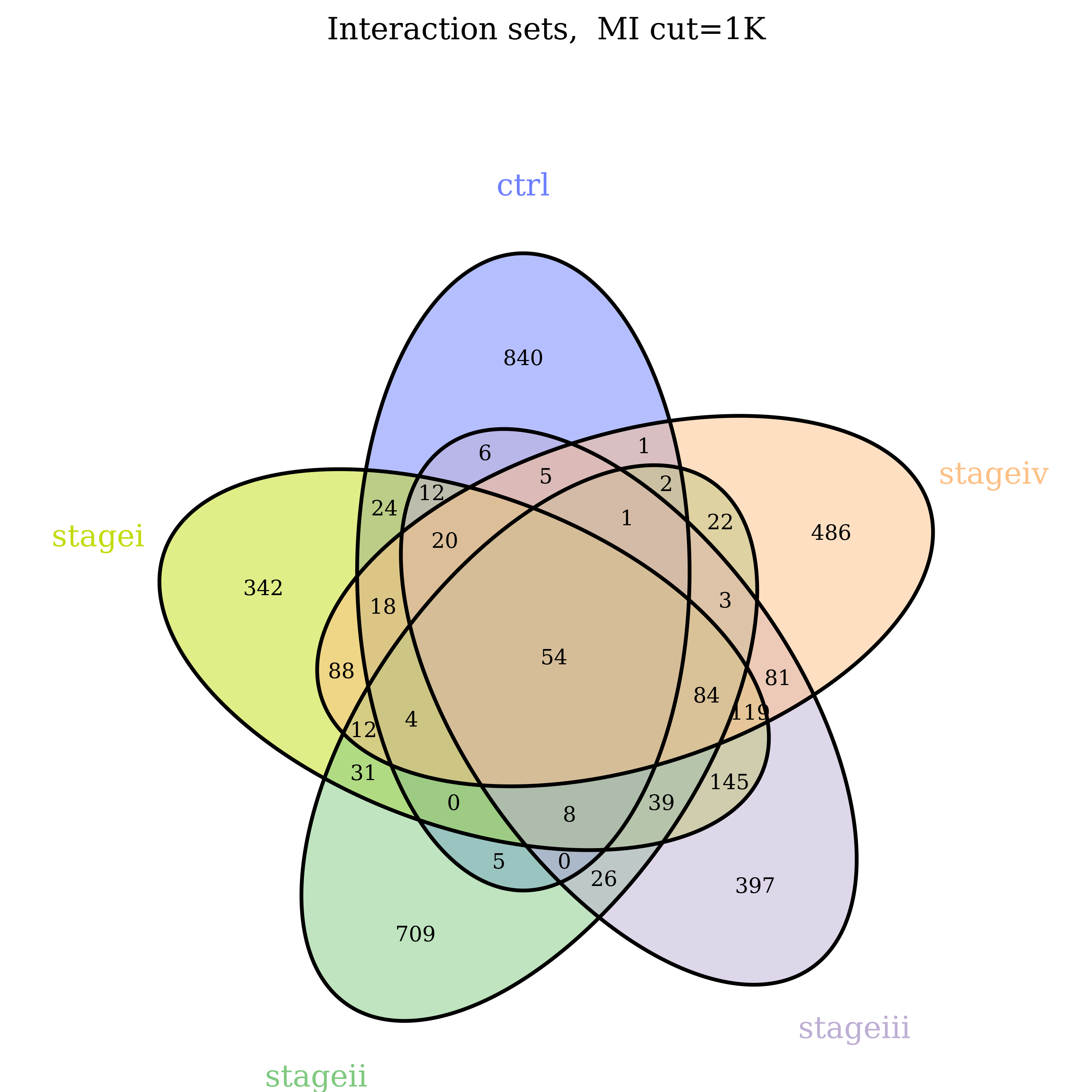

Supplement: Supplementary Material 3 — Heatmaps for intersections and differences in all phenotypes with MI cut-offs of 100, 1,000, 10,000, 100,000, and 1,000,000 interactions. Venn diagrams for intersections of all phenotypes with the aforementioned cut-off values. [file Data_Sheet_3.ZIP › Supp_Mat_3/Venn/venn-1K.png]

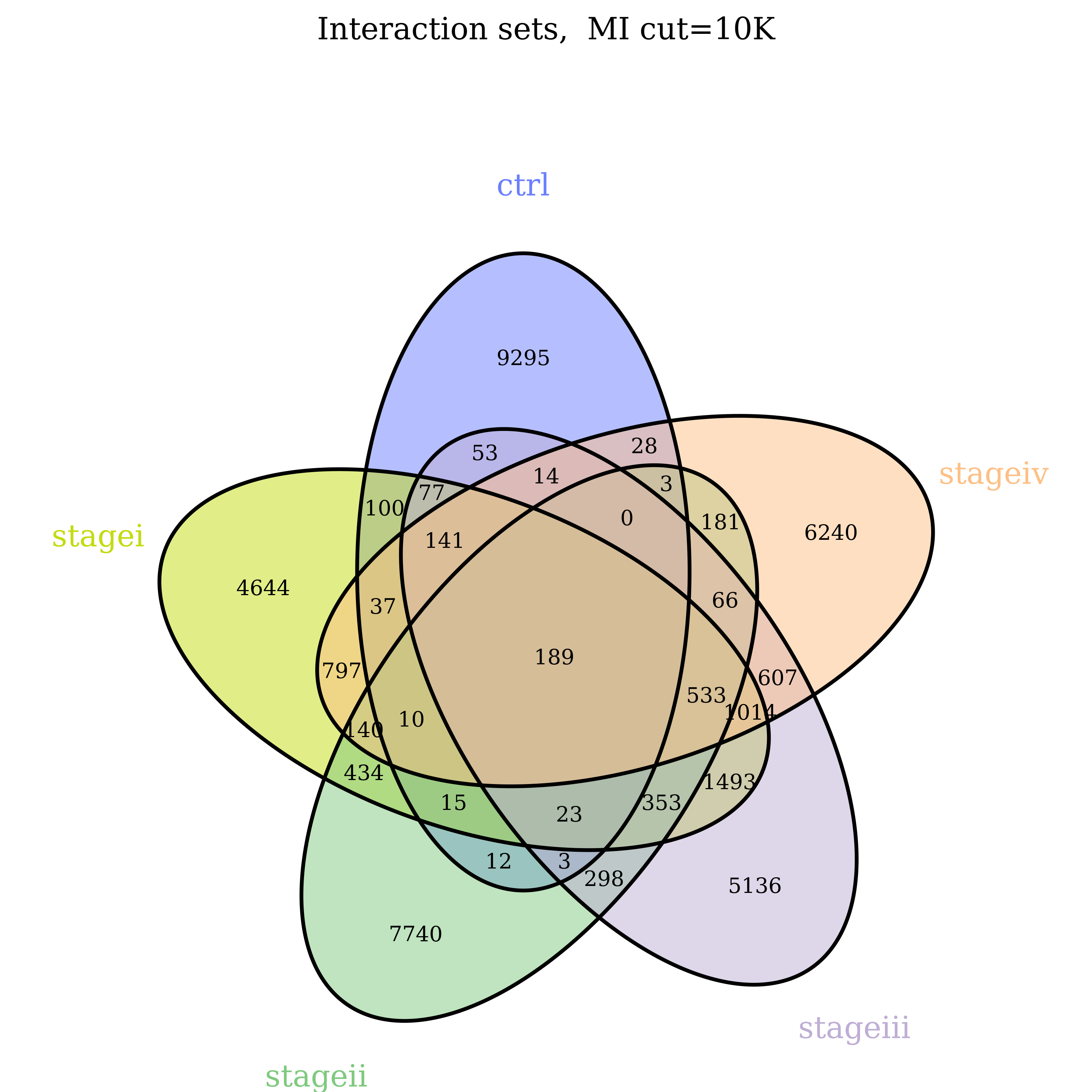

Supplement: Supplementary Material 3 — Heatmaps for intersections and differences in all phenotypes with MI cut-offs of 100, 1,000, 10,000, 100,000, and 1,000,000 interactions. Venn diagrams for intersections of all phenotypes with the aforementioned cut-off values. [file Data_Sheet_3.ZIP › Supp_Mat_3/Venn/venn-10K.png]

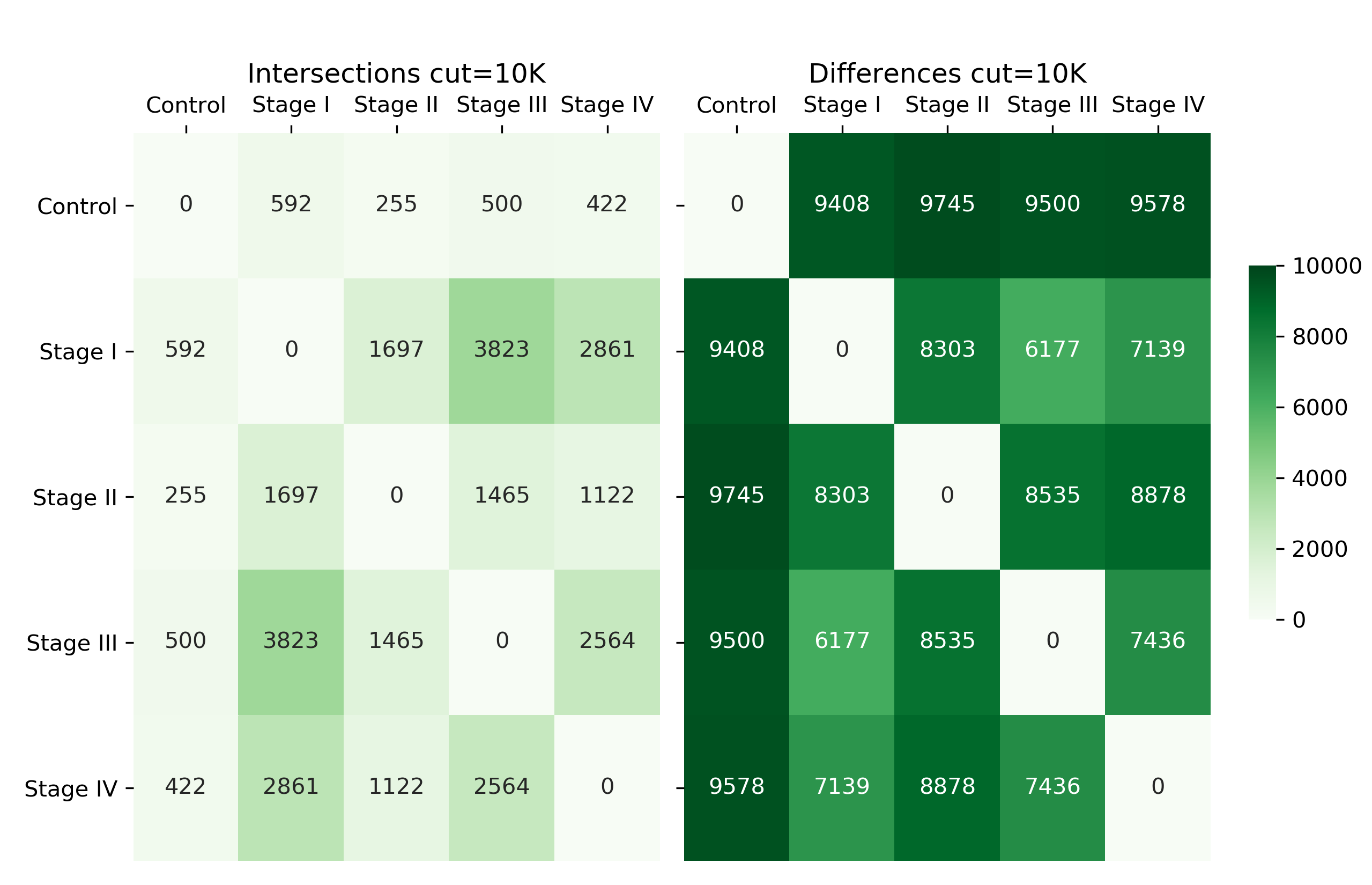

Supplement: Supplementary Material 3 — Heatmaps for intersections and differences in all phenotypes with MI cut-offs of 100, 1,000, 10,000, 100,000, and 1,000,000 interactions. Venn diagrams for intersections of all phenotypes with the aforementioned cut-off values. [file Data_Sheet_3.ZIP › Supp_Mat_3/Heatmaps/heat-interacciones-10K.png]

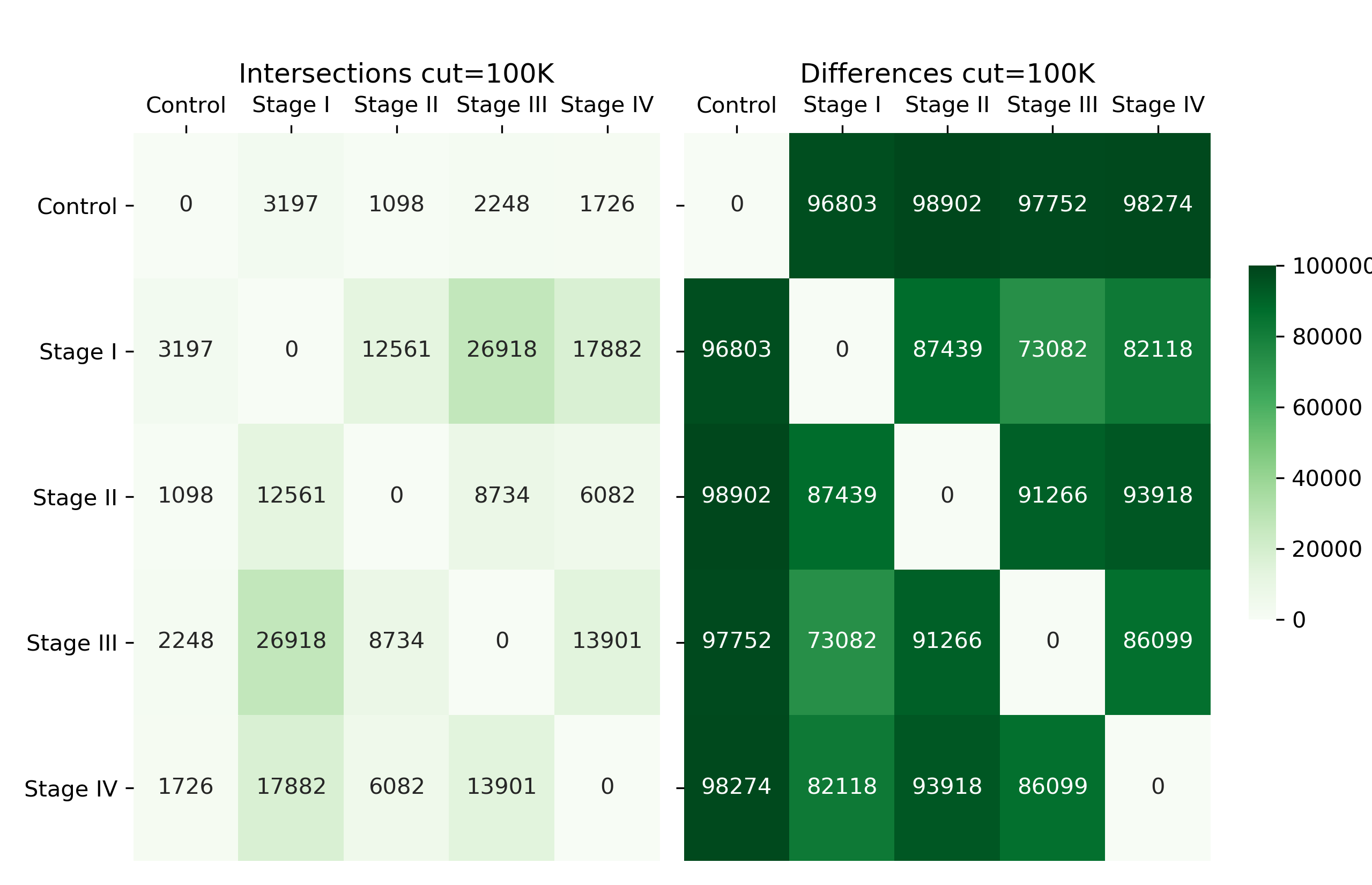

Supplement: Supplementary Material 3 — Heatmaps for intersections and differences in all phenotypes with MI cut-offs of 100, 1,000, 10,000, 100,000, and 1,000,000 interactions. Venn diagrams for intersections of all phenotypes with the aforementioned cut-off values. [file Data_Sheet_3.ZIP › Supp_Mat_3/Heatmaps/heat-interacciones-100K.png]

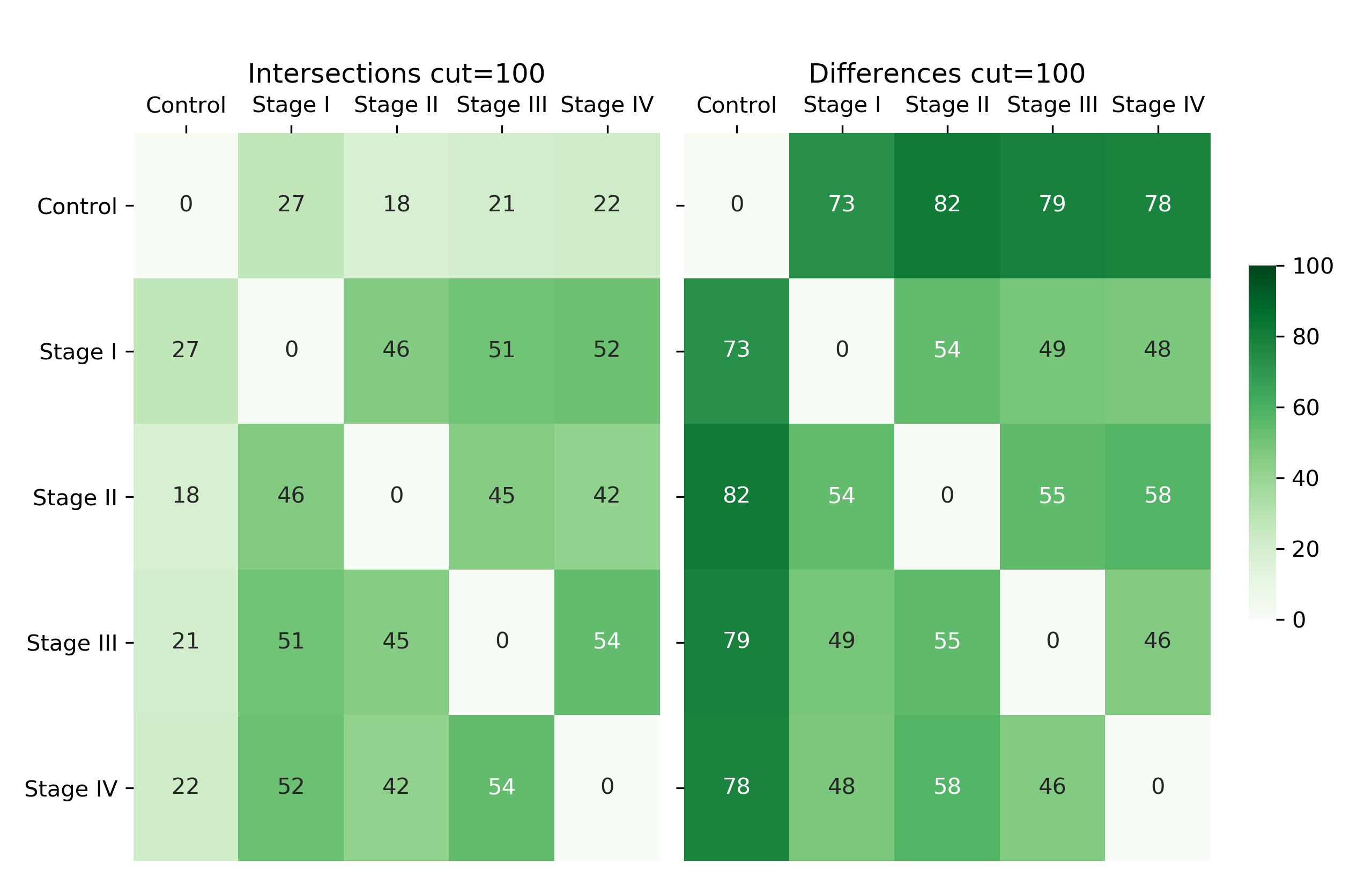

Supplement: Supplementary Material 3 — Heatmaps for intersections and differences in all phenotypes with MI cut-offs of 100, 1,000, 10,000, 100,000, and 1,000,000 interactions. Venn diagrams for intersections of all phenotypes with the aforementioned cut-off values. [file Data_Sheet_3.ZIP › Supp_Mat_3/Heatmaps/heat-interacciones-100.png]

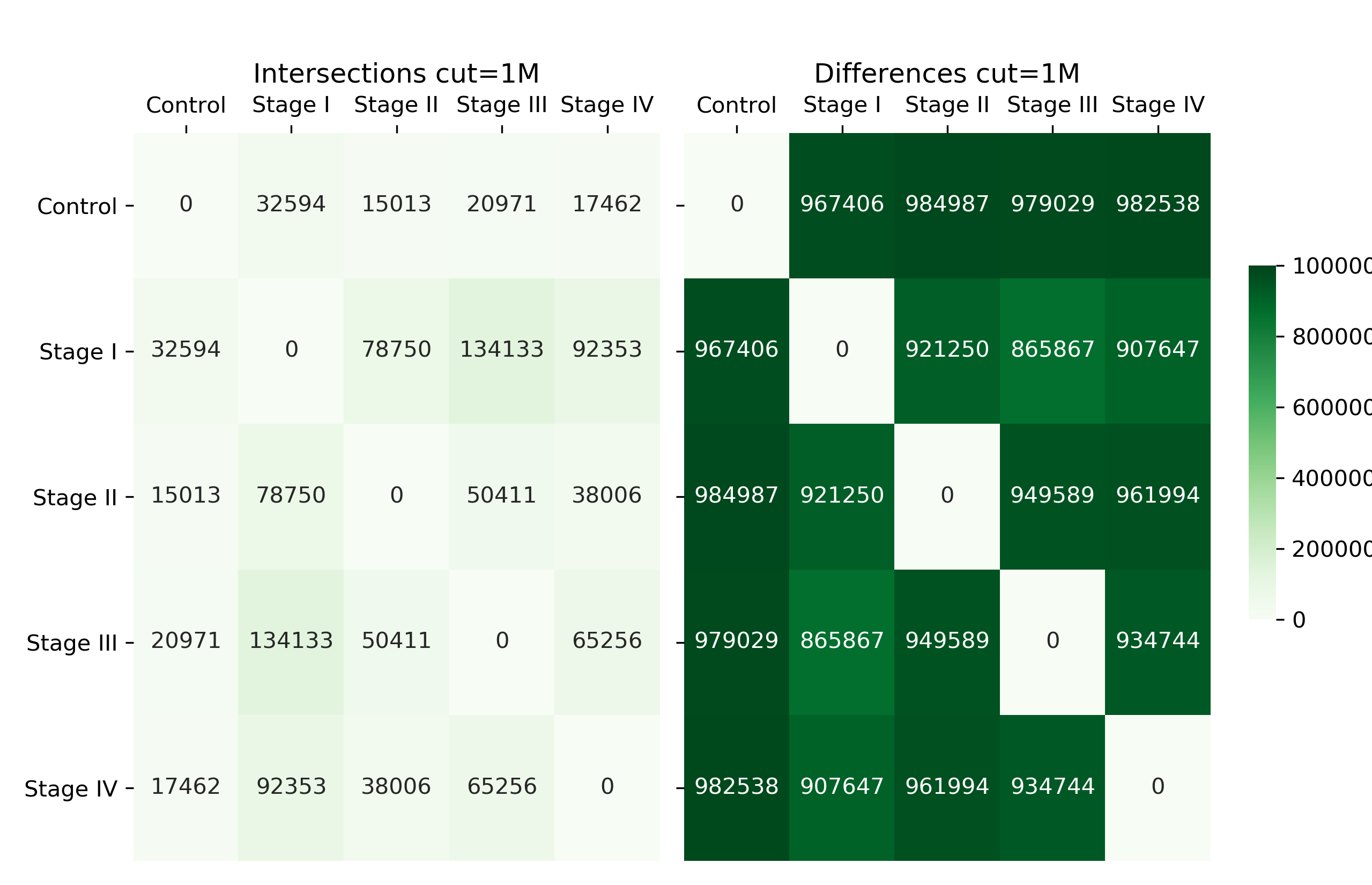

Supplement: Supplementary Material 3 — Heatmaps for intersections and differences in all phenotypes with MI cut-offs of 100, 1,000, 10,000, 100,000, and 1,000,000 interactions. Venn diagrams for intersections of all phenotypes with the aforementioned cut-off values. [file Data_Sheet_3.ZIP › Supp_Mat_3/Heatmaps/heat-interacciones-1M.png]

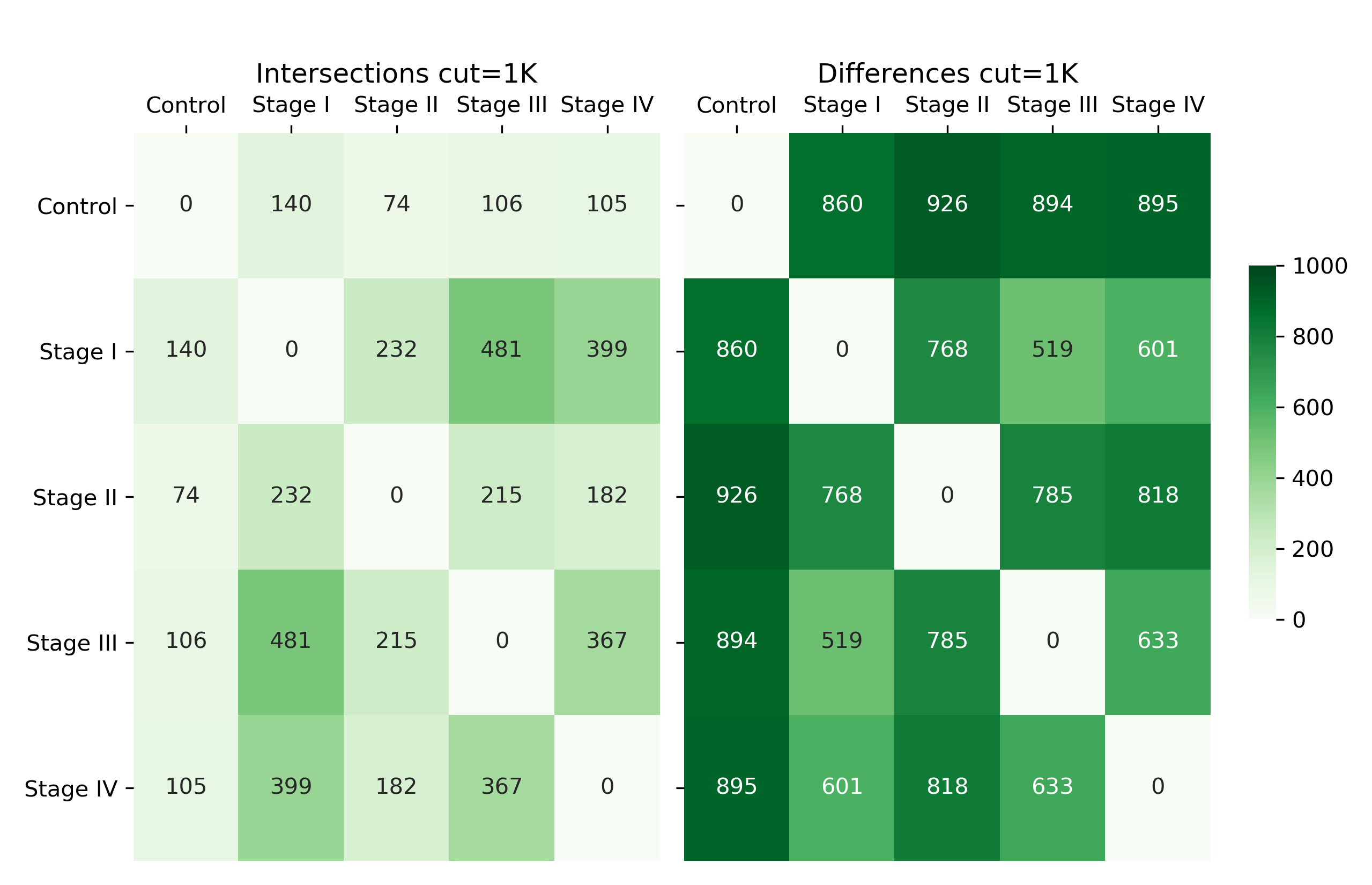

Supplement: Supplementary Material 3 — Heatmaps for intersections and differences in all phenotypes with MI cut-offs of 100, 1,000, 10,000, 100,000, and 1,000,000 interactions. Venn diagrams for intersections of all phenotypes with the aforementioned cut-off values. [file Data_Sheet_3.ZIP › Supp_Mat_3/Heatmaps/heat-interacciones-1K.png]

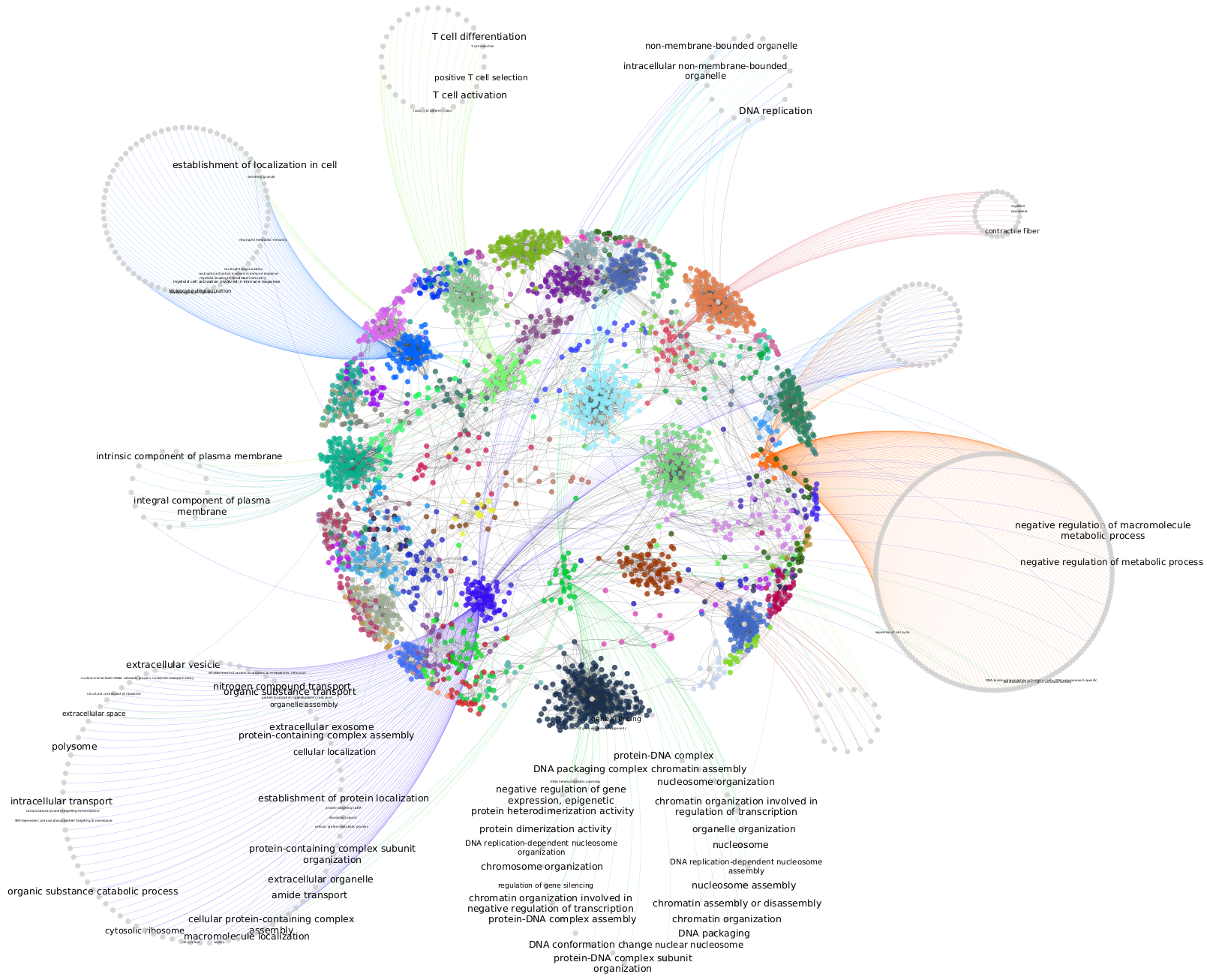

Supplement: Supplementary Material 4 — Complete list of enriched processes for network intersections at different cut-off values. These files contain the enriched categories for both sets, all-phenotypes (control and the four progression stages), as well as ccRC-only intersections. In all cases, the list of enriched categories was performed over network communities, not over the whole geneset. Files are separated based on the cut-off of networks. Visualization of enriched process in Networks of 100,000 and 1,000,000 MI cuts. [file Data_Sheet_4.ZIP › Supp_Mat_4/NETWORK_go_COMM_1M_.png]

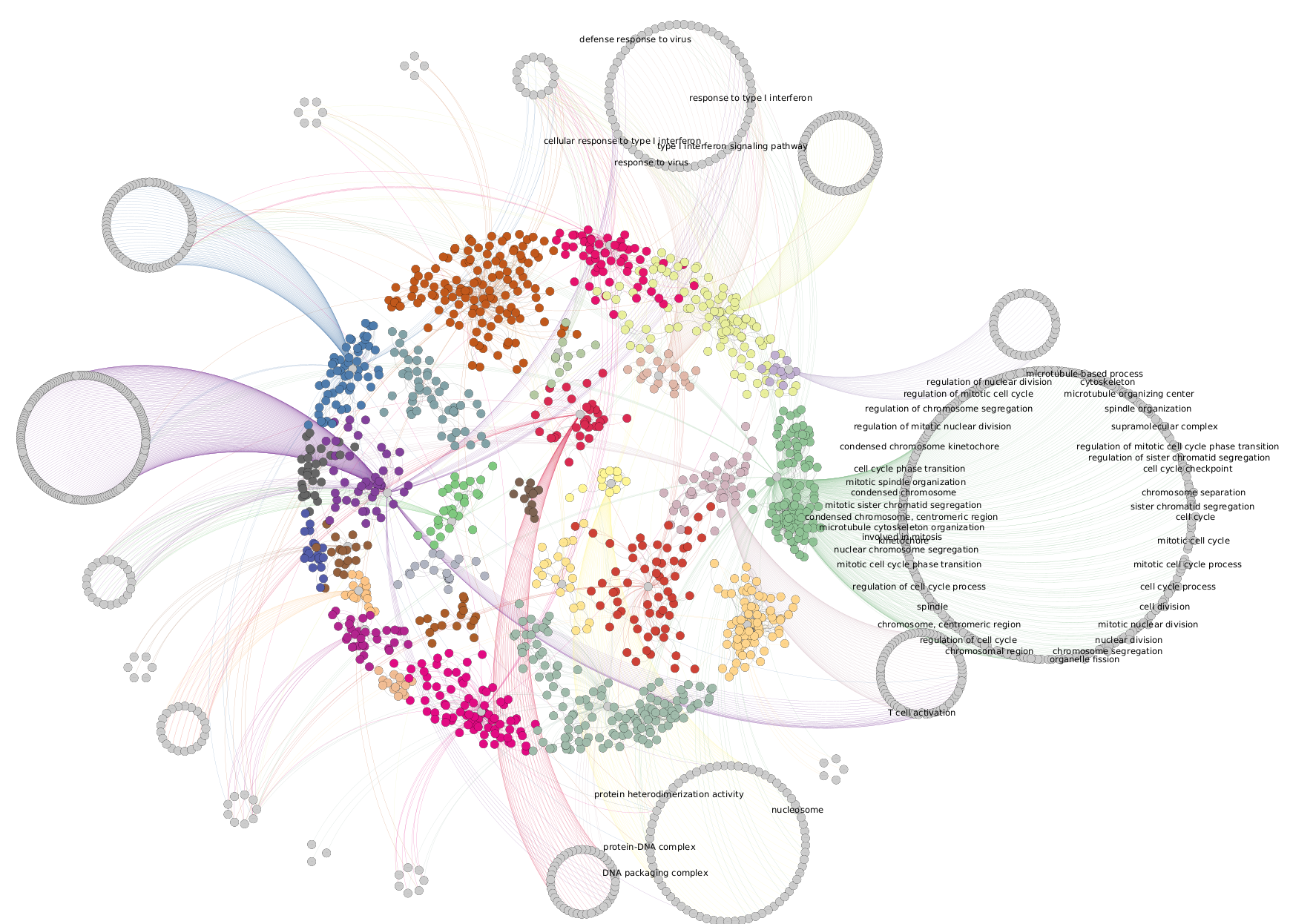

Supplement: Supplementary Material 4 — Complete list of enriched processes for network intersections at different cut-off values. These files contain the enriched categories for both sets, all-phenotypes (control and the four progression stages), as well as ccRC-only intersections. In all cases, the list of enriched categories was performed over network communities, not over the whole geneset. Files are separated based on the cut-off of networks. Visualization of enriched process in Networks of 100,000 and 1,000,000 MI cuts. [file Data_Sheet_4.ZIP › Supp_Mat_4/NETWORK_go_COMM_100K.png]
